# Role of Ag^+^ Ions in Determining Ce^3+^ Optical Properties in Fluorophosphate and Sulfophosphate Glasses

**DOI:** 10.1021/acsomega.1c04933

**Published:** 2021-10-26

**Authors:** Ru Zhou, Courtney Calahoo, Yicong Ding, Lothar Wondraczek

**Affiliations:** Otto Schott Institute of Materials Research, Friedrich Schiller University Jena, Fraunhoferstraße 6, 07743 Jena, Germany

## Abstract

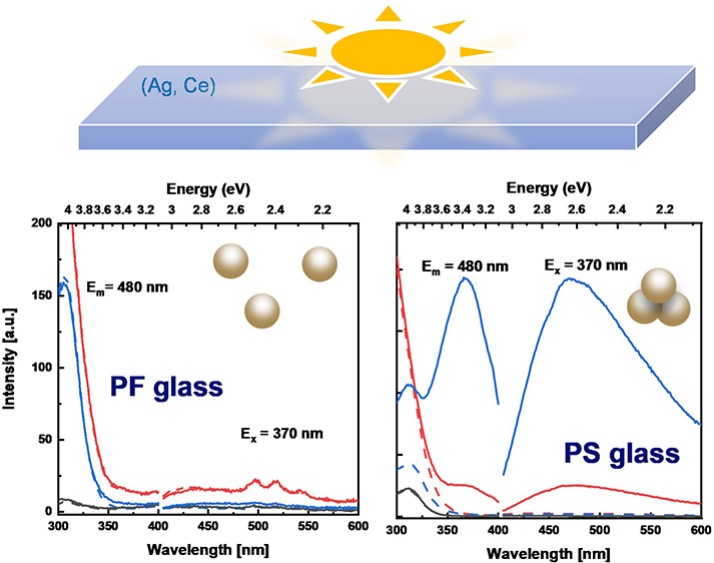

Understanding the interactions among dopant species and the role of the host lattice is of fundamental importance for the chemical formulation of optically active glasses. Here, we consider the archetypal dopant pair of Ag–Ce in complex fluorophosphate (PF) and sulfophosphate (PS) matrices, in which variable bonding environments and ligand selectivity exert distinct effects on dopant properties. The addition of Ag^+^ to PF glasses blue-shifts the ultraviolet (UV) cutoff wavelength of Ce^3+^ and enhances its photoluminescence (PL) intensity. In PS matrices, the exact opposite effect is observed: red-shifting the UV cutoff and lowering the PL intensity. No Ag–Ag pairs or cluster species were found in either matrix material; however, in PS, such clustering could be triggered by secondary broad-band UV–visible irradiation. The optical properties of Ag–Ce-codoped glasses are a result of the ionocovalent character of the Ag^+^–O–Ce^3+^ bond, the cross-relaxation process between Ag^+^ and Ce^3+^, and the redox ratio of Ce^3+^/Ce^4+^. In the PF glasses, the enhancement of the Ce^3+^ PL intensity is due to energy transfer from Ag^+^ to Ce^3+^ and a redox shift from Ce^4+^ to Ce^3+^. The more covalent Ag^+^–O–Ce^3+^ interactions in the PS series decrease the Ce^3+^/Ce^4+^ ratio. Moreover, photoinduced Ag clustering is facilitated in the more covalent environment, which indicates that glasses commonly used for Ag nanoparticle formation, such as silicate glasses, also possess more covalent Ag^+^–O bonding.

## 1 Introduction

Rare earth (RE) ion-activated glasses and fibers are a key component in a variety of optical devices, e.g., for optical telecommunication or fiber lasers.^1−3^ These applications rely on the careful tailoring of the luminescence behavior of active ions via modulating host glass compositions, the state of active species (such as ion valence, spatial distribution, ligand speciation, and coordination number), and potential interactions between active ions (such as energy transfer). Coupling RE ions with Ag nanoparticles is a valuable method to enhance the spectroscopic properties of rare-earth (RE)-doped glasses.^4−6^ In these investigations, the enhanced luminescence was ascribed to the energy transfer from Ag nanoparticles to the RE ions via surface plasmon resonance (SPR). Codoping of cerium into Ag-containing glasses helps to generate Ag molecular clusters and nanoparticles (AgNPs) through UV or X-ray irradiation;^7−9^ the reaction occurring during irradiation is Ce^3+^ + Ag^+^ → Ce^4+^ + Ag^0^. Recently, a study concluded that, unlike large AgNPs that may enhance cerium luminescence by several times, smaller Ag nanoparticles (<50 nm) may quench the Ce^3+^ signal, suggesting atypical energy transfer from Ce^3+^ to Ag^+^.^10^ Indeed, the size, shape, and concentration of the silver nanoparticles were reported to dictate the direction of energy transfer and Ag–Ce interactions in the material.

Although studies often only consider the interactions between metallic Ag^0^ particles and cerium, much of the silver doped into a glass matrix remains as Ag^+^. For example, Simo et al. found 50% of silver to stay as Ag^+^ in soda-lime silicate.^11^ Furthermore, silver may form Ag_m_^*x*+^ clusters with varying amounts of Ag^0^ and Ag^+^ (like Ag^2+^,^12^ Ag_2_^+^,^13^ Ag_3_^2+^,^14^ etc.). The relation between Ag^0^ and Ag^+^ varies easily with temperature, irradiation, and electron donor ability of the glass matrix.^11,12,15^ Despite such considerations, emphasis is usually on the properties of the embedded AgNPs. The optical properties of isolated Ag^+^ ions and interactions between Ag^+^ and further ion species (co-)doped into the glass matrix are much less explored.^3,16^

In a previous report, we discussed Ag doping in fluorophosphate (PF) and sulfophosphate (PS) glass matrices.^17^ Interestingly, for dopant concentrations around 0.5 mol % of Ag and annealing near the glass-transition temperature *T*_g_, no AgNPs or Ag_m_^*x*+^ clusters nor even Ag–Ag pairs were detected in these glasses without additional optical irradiation or secondary heat treatment well above *T*_g_: PF and PS glasses were demonstrated to offer a unique chemical and structural environment within which to explore Ag^+^–RE interactions without the obfuscation from stronger surface plasmon resonance (SPR) of Ag nanoparticles. Furthermore, these investigations revealed the importance of the glass matrix and nearest-neighbor interactions in determining the optical properties of Ag^+^.

In a more general context, phosphate glasses are excellent host materials for optically active ion species;^18−20^ they possess low optical dispersion, low phonon energies, high solubility for many relevant ion species, and low melting points. Aside from cation solubility, phosphate glasses also enable the facile incorporation of secondary anionic species such as sulfate, fluoride, chloride, and nitride ions at high (tens of mol %) concentrations.^21,22^ The addition of such secondary anionic species has pronounced and sometimes unexpected effects on the structure and properties of the phosphate backbone material.^23^ For example, it has been found that the glass-forming ability and corrosion resistance of phosphate glasses can be enhanced significantly through the introduction of sulfate anions (SO_4_^2–^),^24−29^ even though the glass structure was increasingly depolymerized when replacing P_2_O_5_ by SO_3_.^30^ Similar relationships between glass structure and variations in anion content are of interest for understanding the properties of optically active ions doped into such materials, whereby the variation of anion species offers a range of new ligand configurations as compared to that of conventional phosphate glasses.^17,31−34^ Furthermore, anion exchange may reduce the phonon energy such as in the prominent fluoride-phosphate (FP) glasses^35,36^ and may also enhance the transmission window toward the deep blue or ultraviolet (UV) spectral range.^20,37−40^

In the present report, we now investigate the optical properties of cerium in the presence of isolated Ag^+^ only, while comparing the influence of the PF and PS glass’ matrices on the Ag^+^–Ce^3+/4+^ interactions, as well as the effect of irradiation by UV light. The optical absorption, photoluminescence (PL) emission, and excitation spectra were used to study the Ag^+^–Ce^3+/4+^ interactions in the two different matrices, drawing from our previous structural analysis of the base glasses.^17^

## 2 Experimental Section

**Figure 1:**
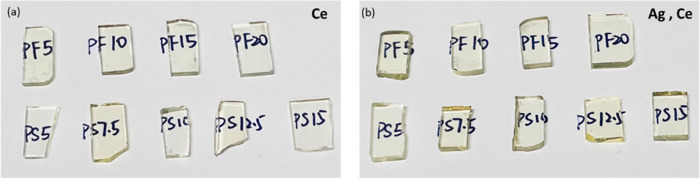
Photographs of (a) Ce-doped PF and PS glasses and (b) Ag,Ce-codoped PF and PS glasses.

Corresponding to a previous study on the role of silver ions in FP and SP glass matrices,^17^ two glass systems with batch compositions (100 – *x*)NaPO_3_-*x*AlF_3_ (*x* = 5, 10, 15, and 20 denoted PF5, PF10, PF15, and PF20, respectively) and (90 – *x*)NaPO_3_-10Al_2_O_3_-*x*Na_2_SO_4_ (*x* = 5, 7.5, 10, 12.5, and 15 denoted PS5, PS7.5, PS10, PS12.5, and PS15, respectively) were studied as host for cerium and silver ion codoping. The glasses were prepared by mixing dried powders in the desired weight fractions. Both glass series, PF and PS, were doped with 0.5 mol % of CeO_2_ or codoped with 0.5 mol % Ag and Ce. High-purity raw materials of AlF_3_, Al_2_O_3_, NaPO_3_, Na_2_SO_4_, AgNO_3_, and CeO_2_ were used. To facilitate the formation of Ce^3+^ (over the oxidized state of Ce^4+^), we added 1 wt % of glucose as a reducing agent. Before melting, all of the raw materials were dried for 4 h at 120 °C. During melting, we kept the melting temperature as low as possible to decrease the fluorine and sulfur losses. We melted 80 g batches in Pt crucibles in a muffle furnace at temperatures between 850 and 1000 °C for 1.5 h (the higher melting temperature was applied at the higher AlF_3_ content in PF glasses, whereas the Al_2_O_3_ content in PS glasses was kept constant). Melts were poured into preheated graphite molds after fining and then annealed between 370 and 420 °C for 3 h before finally cooling to room temperature at a rate of 5 K/min. All glasses obtained in this way were visually transparent without any visible inclusions, bubbles, or striae (shown in Figure 1). The glass slabs were cut to a thickness of 2 mm and polished on both sides for further spectroscopic and mechanical analyses, whereby the latter were performed to confirm material consistency with our previous studies on similar PS and PF glasses, for which we found very good agreement (Table S2 and ref (17)).

Due to significant fluorine and sulfur losses during melting, the actual compositions of PF and PS glasses were determined by energy-dispersive X-ray spectroscopy on a scanning electron microscope (SEM–EDX, JSM-7001F, Japan), as obtained after melting. The results of this analysis are provided in Table S1 for the Ce-doped and Ag,Ce-codoped PF and PS glasses. As discussed previously, confirmation of the fluorine content is further complicated by its low atomic mass, leading to a comparably large error on analyzed fractions.^17,41^

The refractive index *n*_d_ was determined on a Pulfrich refractometer with a precision of ±0.0005. We used a double-beam spectrophotometer (Cary5000, Agilent) with a spectral resolution of 1 nm to record the optical absorption from 800 to 200 nm. PL and PLE spectra were obtained with a spectrofluorometer with double-monochromators in emission and excitation channels (Fluorolog-3, Horiba Jobin-Yvon), using a continuous wave 450 W Xe lamp as a pump source and a Hamamatsu R2658P photomultiplier tube as the detector. A pulsed excitation source (Horiba NanoLed, 309 nm) was used for fluorescence lifetime measurement. Noteworthy, the RE ion density in all of our glasses was relatively high (e.g., ∼8.0 × 10^19^ ions/cm^3^, compared to Ebendorff-Heidepriem et al. using 1.5 × 10^19^ ions/cm^3^).^42^

To investigate the photosensitivity, polished samples with a thickness of 2 mm were irradiated for up to 92 h in a solar simulator equipped with a 1 kW HgXe lamp (Oriel) without further temperature control (this simulator emits a wide spectrum from 230 nm in the UV to the NIR spectral range). The distance between samples and the radiation source was ∼20 cm.

The glass density was determined by Archimedes’ method in dry ethanol to within ±0.001 g/cm^3^. The glass-transition temperature *T*_g_ was obtained from differential scanning calorimetry (DSC, NETZSCH) using a heating rate of 20 K/min under a N_2_ atmosphere. Ultrasonic echography was applied to measure the elastic properties of polished glass samples with a 2 mm thickness. An Echometer 1077 (Karl Deutsch GmbH & Co KG) was used to record the longitudinal and transversal sound wave velocities, *C*_L_ and *C*_T_, at frequencies of 8–12 MHz. The exact specimen thickness was measured with a micrometer screw. Raman spectra were collected using a Renishaw inVia Raman spectrometer (Renishaw; Wotton-under-Edge, U.K.) equipped with an argon ion laser (514 nm excitation and 25 mW power), 2400 lines/mm grating, and a CCD array detector (1024 × 256 pixels). Raman data were collected over the wavenumber range of 200–1500 cm^–1^ with a spectral resolution of ∼2 cm^–1^. The measured Raman spectra were first baseline-corrected and subsequently normalized to the total integrated intensity over the available frequency range (*I*_tot_ = ∫_200_^1500^*I*_mes_(*ω*)d*ω*). All of the Raman spectra obtained in this way are shown in Figures S1–S5. If not otherwise stated, all measurements were carried out at room temperature.

## 3 Results and Discussion

### 3.1 Analyzed Glass Compositions and Physical Properties

Photographs of the as-obtained glass samples are provided in Figure 1. All samples were transparent by visual inspection. The yellowish color indicates the coexistence of Ce^4+^ and Ce^3+^ ions. Results of the chemical analysis are provided in Table S1. The obtained data are in good agreement with those of a previous study in which we used the exact same nominal compositions as host materials for silver ions,^17^ confirming the reproducibility of synthesis procedures in terms of fluoride and sulfate evaporation: the addition of Ag ions into Ce-doped PF and PS glasses had little effect on evaporation loss and physical properties. At the employed melting temperature, melting time, and humidity, we obtain [F]/[Al] of roughly unity, which means that about two-thirds of the batched fluorine evaporated. The sulfur content in the glasses was about half of what was batched.

Additions of fluorine or sulfate to a phosphate glass network have different structural effects. While AlF_3_ may enhance network connectivity, SO_4_^2–^ decreases the degree of polymerization by adding to the lattice in the form of isolated ions charge-compensated by additional Na^+^.^23,43,44^ This reflects in the macroscopic physical properties, where density, Young’s modulus, and *T*_g_ increase with additional AlF_3_ (Table S2). For the PS glass series, Young’s modulus decreases due to a decreasing bond energy density, whereby mass density and *T*_g_ decrease only weakly. Very similar trends have been observed previously;^17,23^ the almost constant value of *T*_g_ was related to the dominant role of Al_2_O_3_.^17^

As mentioned above, Ce^3+^ is very sensitive to the electronic properties of the glass matrix. The capacity of phosphate glasses as host materials for optically active dopant species depends, to a large extent, on the local anionic environments coordinating the doped metal ions.^45^ The electron-donating ability of the anions is determined by the surrounding cations, especially by the glass network formers; this feature can be quantified through the theoretical optical basicity Λ_th_, as introduced by Duffy and Ingram.^46−49^ Lower optical basicity in an oxide glass indicates that the oxygen donates fewer electrons to a doped metal ion and that the bonding between oxygen atoms and other cations is more covalent in the glass. Values for the theoretical optical basicity of the present PS and PF matrix glass series were provided in our previous work.^17^ In both glass series, the optical basicity positively correlates with polarizability and optical refraction.^17,45^

### 3.2 Optical Absorption and Luminescence Properties

#### 3.2.1 Transmittance Spectra

As known from earlier research, the ratio of Ce^3+^/Ce^4+^ mainly depends on the melting temperature and the composition of the glasses.^50−54^ Ce^3+^ and Ce^4+^ are in equilibrium with the dissolved oxygen of the melt according to 4Ce^3+^ + O_2_ ↔ 4Ce^4+^ + 2O^2–^. The equilibrium shifts toward Ce^4+^ with decreasing melting temperature, increasing basicity of the melt, and insufficient reduction capability.

**Figure 2:**
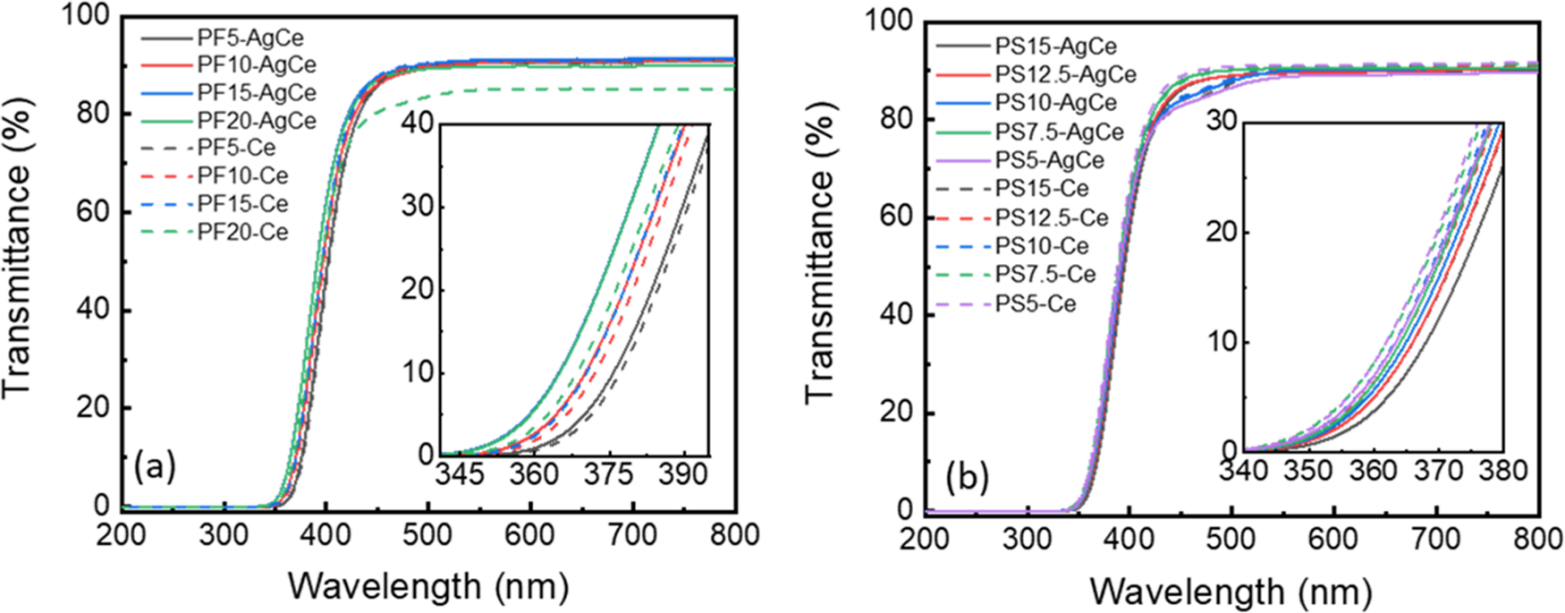
Transmission spectra of (a) Ce-doped and Ag,Ce-codoped PF glasses and (b) Ce-doped and Ag,Ce-codoped PS glasses.

Since both Ce^3+^ and Ce^4+^ ions possess broad near-UV absorption bands, it is difficult to separate the largely overlapping absorption bands of Ce^3+^ and Ce^4+^ ions by spectroscopic methods.^42,55−59^ The transmittance spectra of Ce-doped and Ag,Ce-codoped PF and PS glasses are shown in Figure 2a,b, respectively. All of the glasses exhibit high transmittance in the wavelength range above 450 nm.

The absorption peak of Ce-doped and Ag,Ce-codoped PF and PS glasses at ∼350 nm (Figure S6a,b) is due to the 4f → 5d electronic transitions of Ce^3+^ ions in phosphate glasses.^60,61^ The addition of fluoride in Ce-doped phosphate glass caused a blue shift of the cutoff wavelength from 399 to 392 nm. The addition of sulfate in Ce-doped phosphate glass has a lower influence on the shift of the cutoff wavelength: with increasing sulfate content, the cutoff wavelength shifted from 390 to 385 nm. The blue shift of the cutoff wavelength in PF glasses and the red shift in PS glasses correspond to structural changes in the glass matrix, i.e., variations in the number of nonbridging oxygens in the glass network, which directly affect the matrix’s electron donor capability (varying optical basicity) and the shift of Ce^3+^/(Ce^3+^+Ce^4+^) ratio.^62−67^ As shown in the earlier structural study, the fraction of nonbridging oxygens (NBOs) increases with higher Na_2_SO_4_ and lower AlF_3_ content. The NBOs possess negative charges and are more ionic than the bridging oxygens. Their increasing number causes an increase in the optical basicity of the glass matrix and, thus, a red shift of the cutoff wavelength.

Compared with the absorption spectra of Ag-doped PF and PS glasses in our previous study,^17^ the absorption peak related to the Ag^+^ ion overlaps with the Ce^3+^ absorption in the UV region. As with the Ce-doped glasses, the cutoff wavelength in the Ag,Ce-codoped PF and PS glasses follows the trends in optical basicity of the two glass matrices. Nonetheless, it is worth noting the opposite effect of Ag^+^ in these two series: the cutoff wavelength of Ce^3+^ shifts to the shorter wavelength in Ag,Ce-codoped PF glasses, while the addition of Ag^+^ ions into Ce-doped PS glasses results in a shift to longer wavelength.

The comparisons of transmittance spectra before and after irradiation of Ce-doped and Ag,Ce-codoped PF and PS glasses are displayed in Figure S7. It is very obvious that the transmittance of irradiated samples is smaller than that of the nonirradiated samples. Nonetheless, the absence of a Ag-related absorption peak (expected at ∼400 nm) shows that Ag^0^ clusters or metallic AgNPs do not occur in these samples even after irradiation (Figure S6c,d).

#### 3.2.2 Photoluminescence Properties

**Figure 3:**
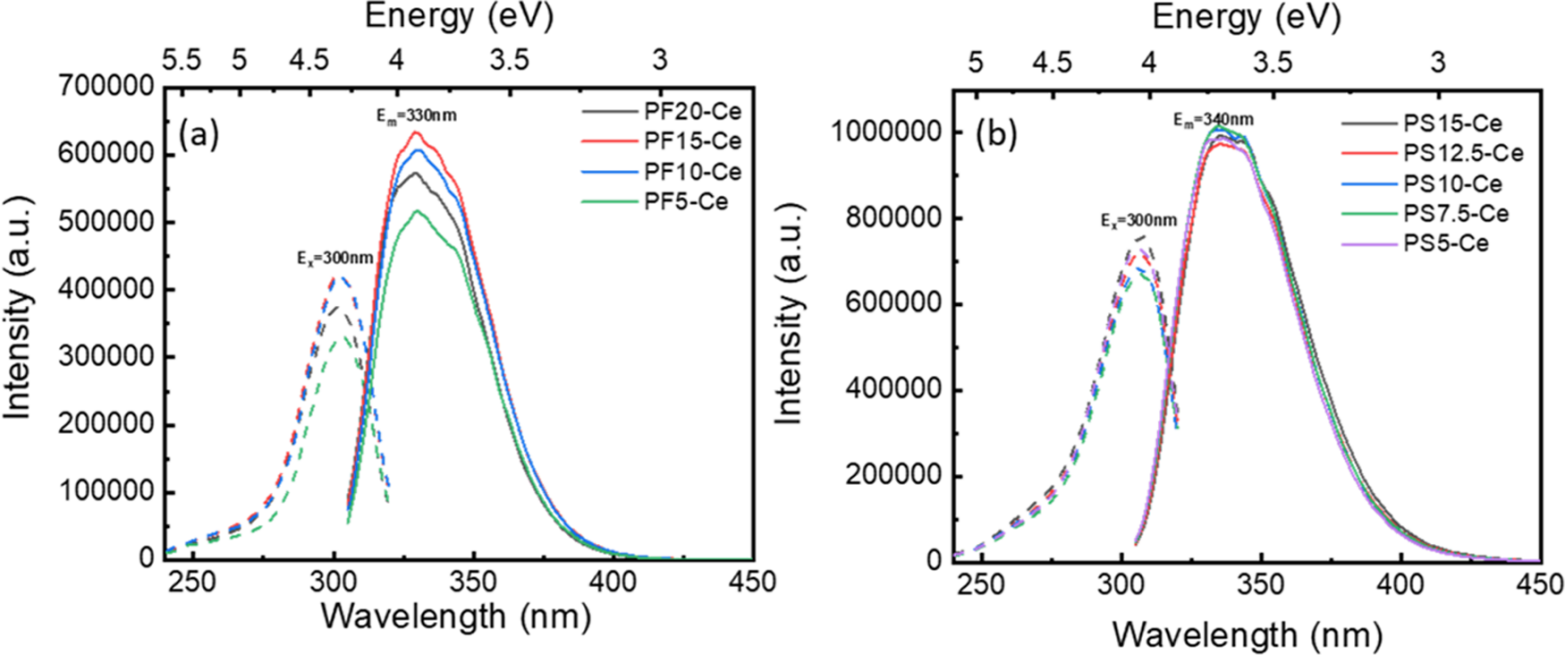
PLE and PL spectra of (a) Ce-doped PF glasses and (b) Ce-doped PS glasses.

The excitation (λ_em_ = 330 nm) and emission spectra (λ_ex_ = 300 nm) of Ce-doped PF and PS glasses are displayed in Figure 3a,b, respectively. Detailed band positions, full width at half-maximum (FWHM), and lifetime information are summarized in Tables 1–4. Unlike Ce^3+^, Ce^4+^ ions show no luminescence due to their closed-shell electronic structure. Thus, the strong and broad asymmetric excitation bands with maxima at ∼300 nm are attributed to the 4f → 5d transitions in Ce^3+^ ions.^68,69^ After being excited to the 5d state by a 300 nm illumination, relaxation to the 4f ground state results in a broad emission band centered at ∼330 nm in Ce-doped PF glasses and 335 nm in PS glasses. The emission center shifts from 334.2 to 336.9 nm with increasing Na_2_SO_4_ and from 329.0 to 331.0 nm with decreasing AlF_3_. The red shift of these Ce^3+^ emission centers corresponds to the increase of optical basicity in the glass network.^62−64^ According to the optical basicity theory, higher optical basicity in glass means an overall increase in the negative charge that Ce^3+^ ions might receive from the surrounding oxygens. Therefore, the electrostatic force of the nucleus on the outermost electrons is weakened, and the electrons are more easily promoted to the 5d energy level.^63^

**Table 1:** Comparisons of Optical Properties of Ce-Doped PF Glasses before and after Irradiation

			emission center		excitation center			
sample	Λ_EDX_	lifetime (ns)	eV	nm	eV	nm	FWHM of emission (eV)	FWHM of excitation (eV)
PF5-Ce	0.454	17.58	3.75	331	4.09	303.2	0.51	0.37
PF10-Ce	0.449	18.14	3.76	330.2	4.10	302.0	0.51	0.38
PF15-Ce	0.447	18.52	3.76	329.7	4.10	302.0	0.50	0.40
PF20-Ce	0.439	18.77	3.77	329.1	4.11	301.7	0.50	0.40
PF5-Ce irr	0.454	16.3	3.75	330.7	4.08	303.9	0.51	0.35
PF10-Ce irr	0.449	17.5	3.76	329.8	4.10	302.4	0.52	0.37
PF15-Ce irr	0.447	17.6	3.77	329.0	4.11	302.0	0.51	0.38
PF20-Ce irr	0.439	18.0	3.77	328.4	4.11	301.7	0.51	0.38

**Table 2:** Comparisons of Optical Properties of Ce-Doped PS Glasses before and after Irradiation

			emission center		excitation center			
sample	Λ_EDX_	lifetime (ns)	eV	nm	eV	nm	FWHM of emission (eV)	FWHM of excitation (eV)
PS5-Ce	0.4525	21.5	3.71	334.2	4.05	306.0	0.53	0.40
PS7.5-Ce	0.4579	21.6	3.70	334.8	4.05	306.2	0.52	0.40
PS10-Ce	0.4583	21.6	3.69	335.6	4.04	306.8	0.52	0.42
PS12.5-Ce	0.4585	21.6	3.69	336.2	4.04	307.0	0.53	0.40
PS15-Ce	0.4589	21.8	3.68	336.9	4.03	307.4	0.53	0.42
PS5-Ce irr	0.4525	20.9	3.68	337.1	4.05	306.0	0.53	0.40
PS7.5-Ce irr	0.4579	20.9	3.67	337.8	4.04	306.9	0.53	0.40
PS10-Ce irr	0.4583	21	3.67	337.8	4.04	307.0	0.53	0.42
PS12.5-Ce irr	0.4585	21.3	3.66	338.6	4.04	307.2	0.54	0.40
PS15-Ce irr	0.4589	21.4	3.66	339.0	4.03	307.7	0.53	0.40

**Table 3:** Comparisons of Optical Properties of Ag,Ce-Doped PF Glasses before and after Irradiation

			emission center		excitation center			
sample	Λ_EDX_	lifetime (ns)	eV	nm	eV	nm	FWHM of emission (eV)	FWHM of excitation (eV)
PF5-Ag,Ce	0.454	17.71	3.75	330.6	4.08	304.0	0.51	0.35
PF10-Ag,Ce	0.449	18.81	3.76	329.6	4.08	304.3	0.51	0.37
PF15-Ag,Ce	0.447	19.96	3.77	328.7	4.09	303.2	0.50	0.38
PF20-Ag,Ce	0.439	19.56	3.78	328.4	4.10	302.4	0.50	0.37
PF5-Ag,Ce irr	0.454	16.9	3.73	332.4	4.08	303.9	0.51	0.35
PF10-Ag,Ce irr	0.449	18.4	3.74	331.5	4.11	302.0	0.51	0.37
PF15-Ag,Ce irr	0.447	19.5	3.76	330.2	4.11	301.8	0.51	0.38
PF20-Ag,Ce irr	0.439	19.4	3.76	329.5	4.11	301.6	0.50	0.38

**Table 4:** Comparisons of Optical Properties of Ag,Ce-Doped PS Glasses before and after Irradiation

			emission center		excitation center			
sample	Λ_EDX_	lifetime (ns)	eV	nm	eV	nm	FWHM of emission (eV)	FWHM of excitation (eV)
PS5-Ag,Ce	0.4525	20.4	3.71	334.5	4.05	306.0	0.52	0.42
PS7.5-Ag,Ce	0.4579	20.5	3.70	335.4	4.04	306.8	0.52	0.40
PS10-Ag,Ce	0.4583	20.6	3.69	336.4	4.04	307.0	0.52	0.42
PS12.5-Ag,Ce	0.4585	20.6	3.68	336.8	4.04	306.6	0.53	0.40
PS15-Ag,Ce	0.4589	21.3	3.67	337.6	4.03	307.8	0.53	0.40
PS5-Ag,Ce irr	0.4525	20.0	3.67	337.6	4.05	306.0	0.52	0.40
PS7.5-Ag,Ce irr	0.4579	20.4	3.67	337.6	4.04	306.9	0.53	0.40
PS10-Ag,Ce irr	0.4583	20.4	3.67	337.6	4.04	306.6	0.53	0.42
PS12.5-Ag,Ce irr	0.4585	20.4	3.66	339	4.03	307.5	0.54	0.40
PS15-Ag,Ce irr	0.4589	20.9	3.65	340.1	4.03	308.0	0.53	0.40

Although an increase in optical basicity has a positive correlation with the emission peak position in the Ce-doped glass compositions, the magnitudes of the peak shifts are unexpected: the Λ_th_ changes noticeably more in the PF series compared to the variation in Λ_th_ in the PS series, yet the shifting of the PF luminescence peak is smaller than that of the PS luminescence peak. A glass system usually contains more than just a single polarized state, such as the bridging oxygens (lower polarizability) and nonbridging oxygens (higher polarizability), and they each have different optical basicities. Therefore, the calculated value of Λ_th_ for the glass is an average value throughout the material. The NMR results of our previous structural study indicated that there are more *Q*^1^ and *Q*^0^ species in PS glasses than in PF glasses.^17,23^ The transformation of *Q*^2^ species to *Q*^1^ species with an increasing NaSO_4_ content is evident. Considering the structural analysis, the trend of peak shift indicates that Ce^3+^ is more responsive to the electronic environment of sulfophosphate glasses than to that of fluorophosphate glasses because of the higher concentration of *Q*^1^ and *Q*^0^ species in the PS matrix.

**Figure 4:**
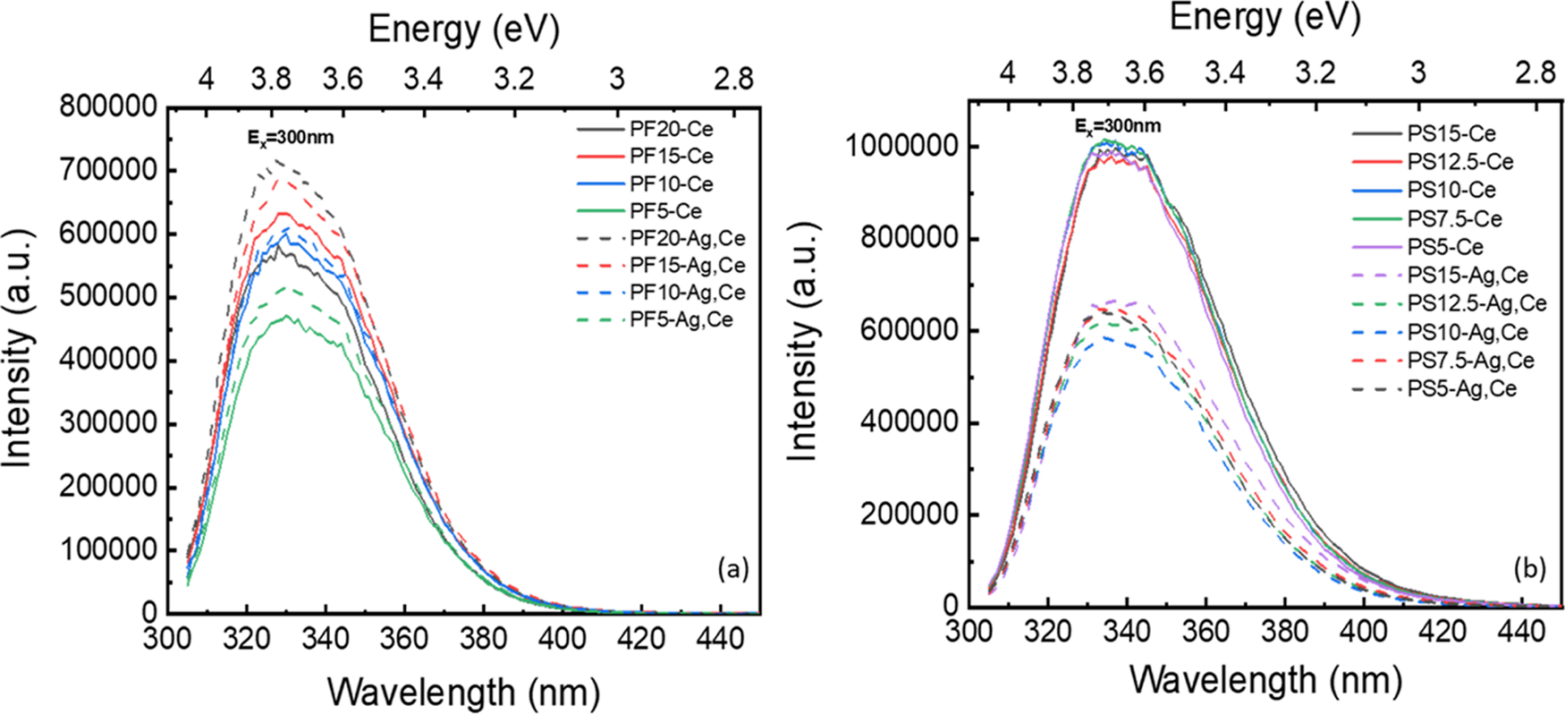
Comparisons of PL spectra of Ce-doped and Ag,Ce-codoped (a) PF glasses and (b) PS glasses.

The PL and PLE spectra of Ag,Ce-codoped PF and PS glasses are presented in Figure S8a,b. Since the absorption peak of silver ions is overlapped by Ce^3+^ in Ag,Ce-codoped glasses, there are no obvious observations of the emission band of Ag^+^ in the codoped glasses. It follows that the PLE and PL spectra of the Ag,Ce-codoped PF and PS glasses show a similar emission and excitation peak as the Ce-doped PF and PS glasses. Paradoxically, the introduction of Ag^+^ to PF glasses increases the emission intensity of Ce^3+^, while the same Ag^+^ addition reduces the intensity of emission in PS glasses (shown in Figure 4). The addition of Ag^+^ has substantial effects on the PL intensity, demonstrating that Ag^+^ ions without the formation of Ag nanoparticles can induce a large variation in the optical properties of cerium and possibly other RE ions.

**Figure 5:**
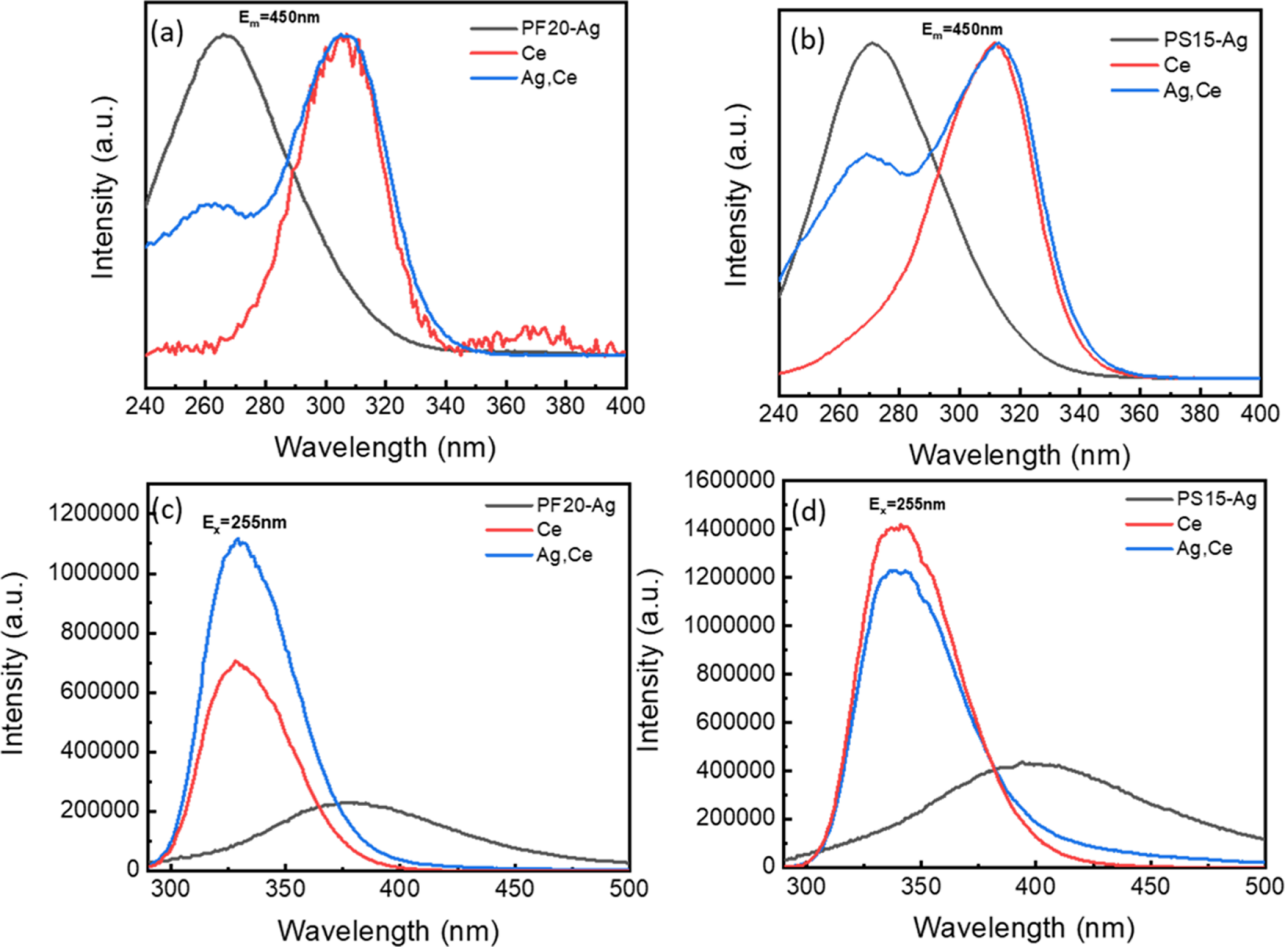
PLE spectra of (a) PF20 and (b) PS15 glasses under *E*_m_ = 450 nm; PL spectra of (c) PF20 glass and (d) PS15 glass.

In Figure 5, we compare the PLE and PL spectra of Ag-doped, Ce-doped, and Ag,Ce-codoped PF20 and PS15 glasses under an emission wavelength of 450 nm and an excitation wavelength of 255 nm, respectively. The PLE spectra and PL spectra are similar also for all of the other studied compositions of PF and PS glasses. It is very obvious that the emission spectra of Ag,Ce-codoped glasses have two excitation centers located at ∼260 and ∼307 nm. The 260 nm excitation peak belongs to the Ag^+^ and the 307 nm excitation peak is related to the Ce^3+^. When we use 255 nm to excite the Ag,Ce-codoped glasses, the emission spectra are analogous with the corresponding Ce-doped PF and PS glasses, but because of the overlap of the Ag^+^ band by the Ce^3+^ emission band, we must deconvolute with Gaussian functions to resolve the hidden Ag^+^ emission band (Figure 11). Figure 5 indicates that the variation in the PL intensity of Ce^3+^ codoped with Ag in these glasses may be due to energy-transfer processes between Ag^+^ and Ce^3+^, which is discussed in Section 3.3. Furthermore, the redox equilibrium of Ag might be the reason for the weaker intensity of the Ag^+^ excitation band in Ag,Ce-codoped glasses. Moreover, the variations of the cerium PL intensity may also be due to the variation of the valence of the cerium ion and associated Ag–Ce interactions.

#### 3.2.3 Effect of Irradiation on Photoluminescence and Lifetime

**Figure 6:**
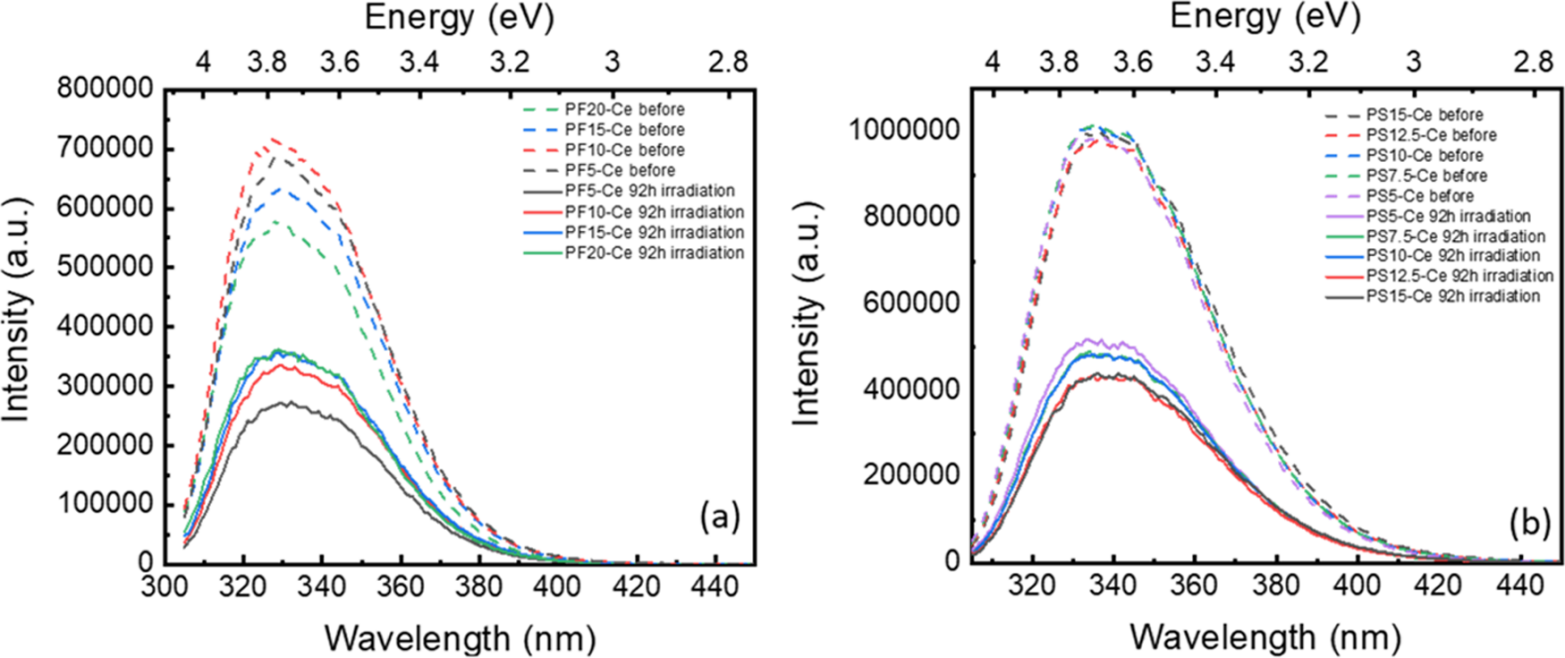
Emission spectra of Ce-doped (a) PF glasses and (b) PS glasses before and after irradiation under *E*_x_ = 300 nm.

Comparisons of optical properties of Ce-doped and Ag,Ce-codoped PF and PS glasses are summarized in Tables 1–4. After irradiation, the PL intensity and the lifetime of Ce^3+^ decrease in all compositions. Furthermore, a new emission band related to Ag clusters can be observed in Ag-doped and Ag,Ce-codoped PS glasses. Figure 6a,b shows the emission spectra of Ce-doped PF and PS glasses before and after 92 h of irradiation, respectively. It is very evident that after irradiation, the PL intensities of Ce^3+^ of both PF and PS glasses decrease. Identical behavior is observed in Ag,Ce-codoped PF and PS glasses (Figure S9).

**Figure 7:**
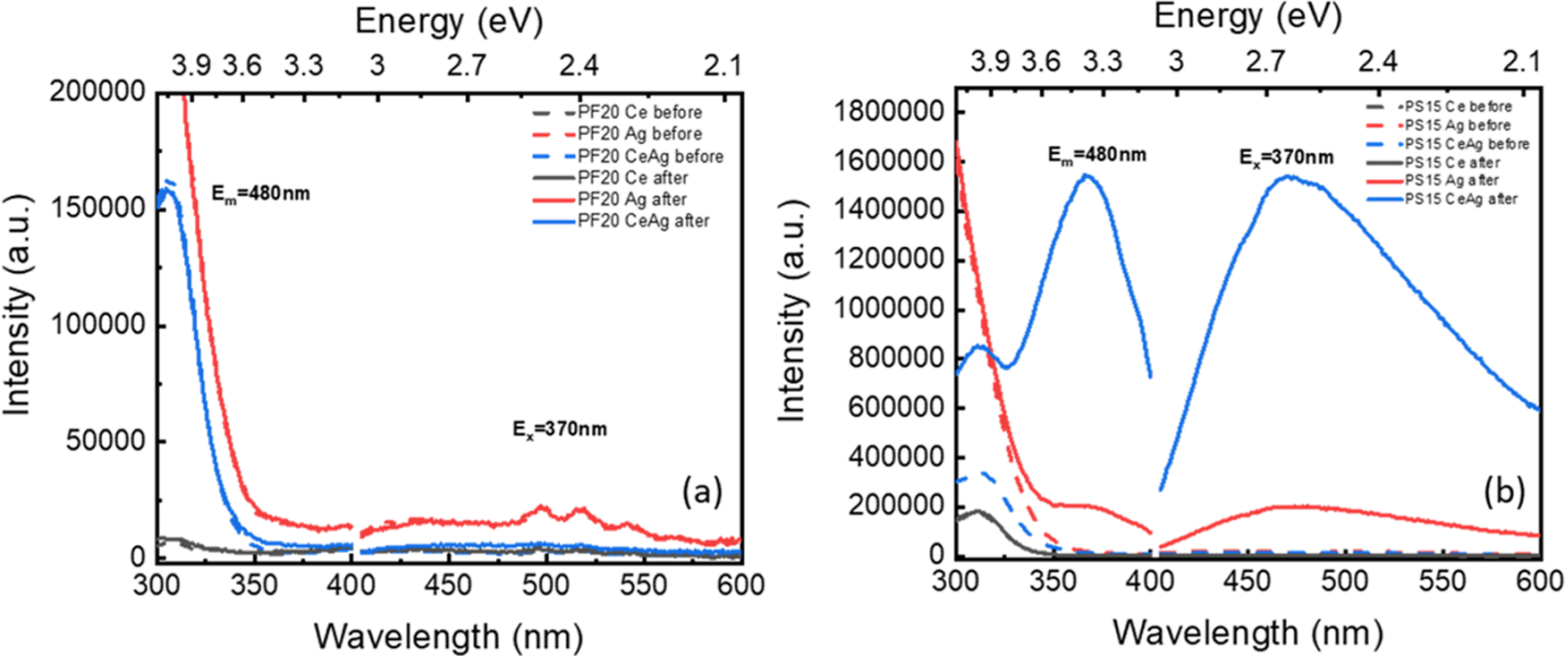
PL and PLE spectra of (a) PF20 and (b) PS15 glasses under *E*_x_ = 370 nm and *E*_m_ = 480 nm before and after irradiation.

Figure 7 displays the PL (*E*_x_ = 370 nm) and PLE (*E*_m_ = 480 nm) spectra of Ag-doped, Ce-doped, and Ag,Ce-codoped PF20 and PS15 glasses before and after irradiation. Here, because the optical behavior of PF and PS glasses are similar, we only present the PL and PLE spectra of PF20 and PS15 glasses. From Figure 7, a new emission peak at ∼480 nm appears in Ag-doped PS and Ag,Ce-doped PS glasses after irradiation (the PL and PLE spectra before and after irradiation are unchanged in PF glasses). According to the literature, the emission peak centered at 480 nm corresponds to Ag_m_^*x*+^ clusters composed of Ag^+^ ions and neutral Ag^0^.^14,70^ The same phenomenon was also found in Ag,Ce-codoped zinc–sulfophosphate glass after UV irradiation.^9^ The results reveal that PS glasses exhibit a higher ability to generate Ag clusters during irradiation as compared to the PF glasses.

**Figure 8:**
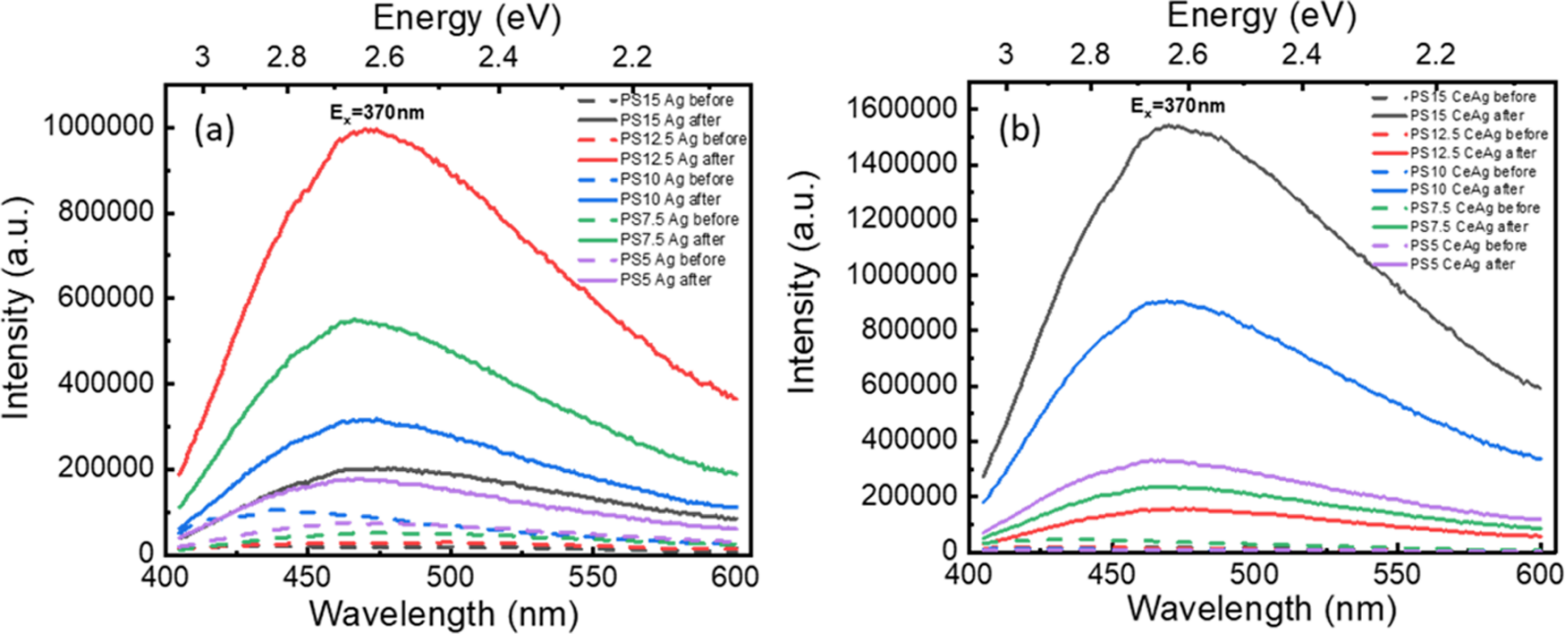
PL spectra of (a) Ag-doped PS glasses and (b) Ag,Ce-codoped PS glasses before and after irradiation.

Figure 8 shows the PL intensity of the Ag-doped and Ag,Ce-codoped PS glasses under excitation at 370 nm. After irradiation, the emission intensity of Ag_m_^*x*+^ clusters is significantly enhanced. However, no clear relation between the sulfate content and the PL intensity of the Ag cluster after irradiation is obtained from Figure 8. Comparing the Ag_m_^*x*+^ cluster intensity between Figure 8a,b (Ag only and Ag–Ce) before irradiation, the cerium may prevent Ag_m_^*x*+^ cluster formation, while cerium clearly promotes Ag_m_^*x*+^ cluster formation during irradiation (as the intensity is much higher in Ag–Ce than in Ag alone after irradiation).

**Figure 9:**
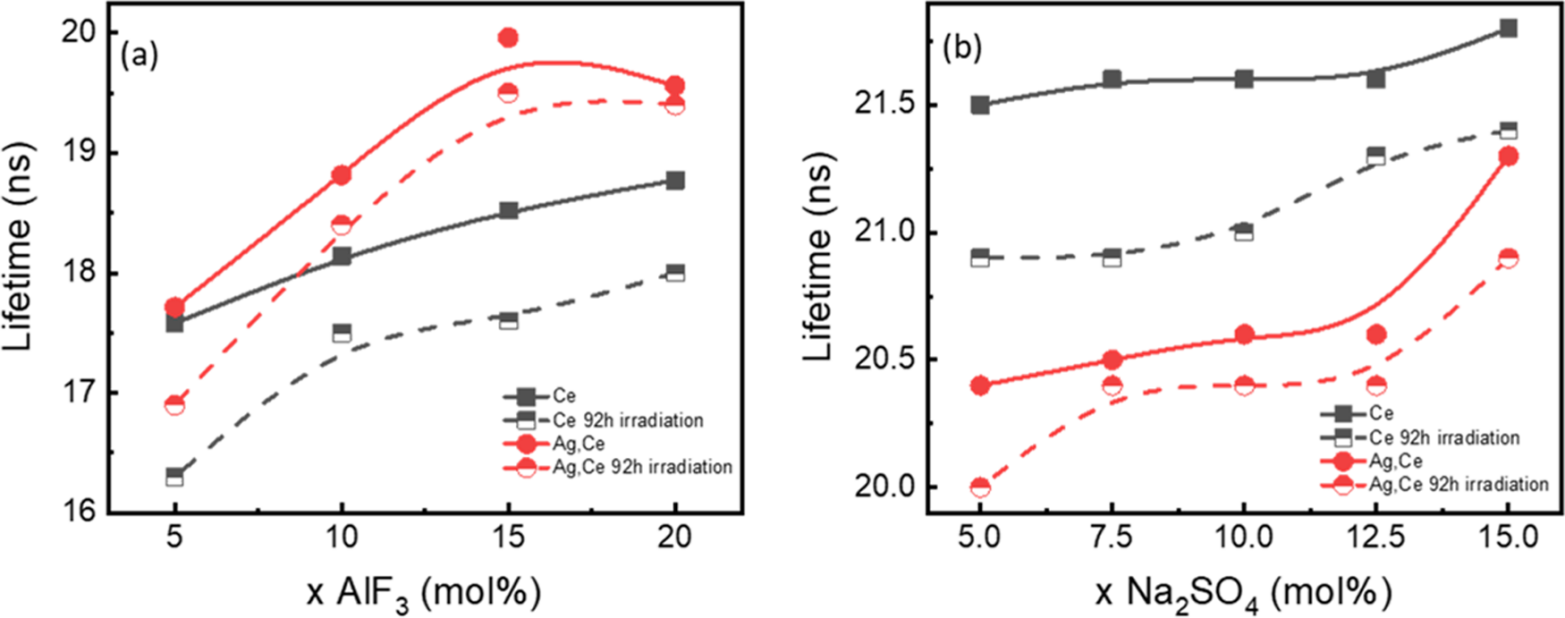
Lifetime of Ce-doped and Ag,Ce-codoped (a) PF glasses and (b) PS glasses before and after irradiation under the *E*_x_ = 309 nm and *E*_m_ = 330 nm.

The luminescence decay curves of the Ce-doped PF and PS glasses are depicted in Figure S10. The lifetimes of emission at 330 nm are deduced by an approximation with a double-exponential decay function. The average lifetime of the emission at 330 nm under an excitation of 309 nm became longer with higher fluoride but stays almost constant with an increasing sulfate content: in PF glasses, it grew from 17.6 to 18.8 ns, and in PS glasses, it stays at ∼21.5 ns. The obtained lifetimes are close to values typically reported for Ce^3+^ photoluminescence from silicate matrix glasses, i.e., varying within ∼15–30 ns.^66,71^ The longer lifetime of PS glasses might be because of the variation in phonon energy obtained by the addition of sulfate.^17^ The lifetime of the Ce^3+^ emission at 330 nm under an excitation of 309 nm in PF and PS glasses, with and without Ag^+^, as well as before and after irradiation, is plotted in Figure 9. The trends of the lifetime of Ag,Ce-codoped glasses are the same as those of the Ce-doped glasses. Nevertheless, when Ag is introduced, the glass networks of the PF and PS series have different effects on the decay time of Ag,Ce-codoped glasses. In PF glasses, codoping with Ag^+^ enhances the lifetime of Ce^3+^, while Ag^+^ in Ce-doped PS glasses slightly reduces the lifetime of Ce^3+^. As expected, due to the conversion of Ce^3+^ to Ce^4+^ during the irradiation, the lifetimes of all of the Ce-containing glasses after irradiation are lower than the lifetime before the irradiation.^72^ Additionally, comparing Figure 6 with Figure S9, the reduction of the PL intensity of Ce^3+^ is weaker after irradiation even in the presence of Ag^+^ ions in both PF and PS glasses.

### 3.3 Influence of Ag^+^ Ions on Ce^3+^ Optical Properties

Like the optical probes used by Duffy and Ingram (Pb^2+^, Tl^+^, Bi^3+^),^73^ the interaction between Ce^3+^ and oxygen is such that the optical trends for Ce^3+^ have the expected relationship with both the average and local optical basicity of the glass matrix, as determined by the amount of *Q*^0^ and *Q*^1^ units in the PF and PS series. In an earlier paper, we reported how the increase of AlF_3_ in PF and Na_2_SO_4_ in PS glasses has opposite effects on the glass structure, with AlF_3_ reducing the number of highly charged *Q*^0^ + *Q*^1^ units and PS increasing the number of *Q*^0^ + *Q*^1^ units.^17^ Ce^3+^ has a field strength of 0.53 Å^–2^, putting it strictly in the intermediate category beside tetrahedral Mg^2+^, thereby agreeing with studies showing that the Ce–O bond has a substantial covalent character in both CeO_2_ and Ce_2_O_3_.^74,75^ In our previous work, we argued that only when silver is behaving covalently does it agree with Duffy’s optical basicity theory, which is in line with the theory fitting cerium here. Currently, we do not have adequate models to describe the optical trends of categorical glass modifiers, such as Na^+^ and Ca^2+^, whose interaction with molecular orbitals of NBOs appears to be different.

Since the trend for the cerium absorption cutoff in the PS series is smaller than that in the PF glasses, cerium does not appear to preferentially associate with sulfate, and like Ag^+^, may be more strongly associated with *Q*^0^ and *Q*^1^ units. The number of *Q*^0^ + *Q*^1^ units is relatively high in all of the PS glasses, while they decrease substantially with increasing AlF_3_ concentration. The same association preference was observed for sulfophosphates, with Zn^2+^ and Na^+^ being associated with PO_4_^3–^ and SO_4_^2–^ ions, respectively.^25^

Despite optical basicity and structural trends fitting adequately for Ce^3+^, this does not explain directly the fact that silver has opposite effects on cerium’s optical properties when introduced into our glasses. The *Q^n^*-distribution or number of NBOs is not expected to change largely from only a 0.5 mol % Ag_2_O addition (shown in Figures S3–S5), yet some of the variations in Ce^3+^ optical properties are larger upon silver introduction than the stepwise addition of Na_2_SO_4_. As such, we believe the silver to be strongly associated with cerium in the glass to have such a direct influence. This is perhaps an unsurprising conclusion, given the large amounts of literature discussing Ag–Ce interactions and the long history of these two ions in photography.^7,10,76^

During this entire discussion, we must always remember the equilibrium 4Ce^3+^ + O_2_ ↔ 4Ce^4+^ + 2O^2–^, which may compete with the corresponding equilibrium for silver, 4Ag^0^ + O_2_ ↔ 4Ag^+^ + 2O^2–^. Unfortunately, it is very challenging to quantify the amount of Ce^3+^/Ce^4+^ when Ce^3+^ is the majority oxidation state, with XPS, EPR, and optical spectroscopy all having their unique challenges.^42,65,77^ Since the broad charge-transfer band of the Ce^4+^ has been observed to shift the absorption edge of cerium-containing glasses into the visible range, converting colorless glasses to yellow, the absorption edge of the codoped glasses here may also be correlated with the amount of Ce^3+^/Ce^4+^.^42^ Recently, Cicconi et al. determined the Ce^3+^/Ce^4+^ redox ratio in silicate glasses/melts using a multispectroscopy method.^78^ However, in our present case, we do not see a consistent Ce^4+^ tail to emerge when we add silver in Figure S6, and since we add a reducing agent to the melt (1 wt % sugar), we do not believe the addition of silver to substantially alter the cerium oxidation state. Furthermore, the addition of Ag^+^ would be expected to influence the cerium equilibrium in the same direction in both glass series, revealing the impact of the PS vs PF glass matrix.

Although the two equilibria share products/reactants, we can only assume that the *K*_eq_(Ce) ≪ *K*_eq_(Ag), as these glasses seem to disproportionately stabilize Ce^3+^ and Ag^+^, with only small amounts of Ce^4+^ being suspected in PS7.5–PS15 (Figures 2 and S6) and Ag_m_^*x*+^ clusters only forming after irradiation in PS (see Figure 8b). The PF glasses do not appear to ever form Ag_m_^*x*+^ clusters or Ag^0^, even after irradiation (Figure 8a); thus, the equilibria in these glasses appear to heavily favor one side. Since the first-electron reduction potentials of Ag^+^ and Ce^4+^ are 0.7996 and 1.61 V,^79^ respectively, it follows that we have no discernible Ce^4+^ in our glasses, yet can simultaneously have majority Ag^+^.

The PL and PLE spectra in Figure 7 reveal that after irradiation the PS matrix facilitates some silver-clustering and Ag^0^ formation, even more so in the presence of Ce. Based on our previous structural studies of these glasses, the Ag^+^ associates preferentially with *Q*^0^ + *Q*^1^ units and “removes” oxygen from the phosphate network. Furthermore, we discussed that one of the possible steps before silver reduction is the formation of covalent Ag_2_O-like regions, which is facilitated by the presence of *Q*^0^ and *Q*^1^ units in the phosphate network. The structural environment in PF glasses is unique in that it appears to strongly discourage Ag clustering and Ag^0^ formation, where other common glass networks (such as silicates) immediately form silver nanoparticles during melt-quenching or annealing.^11,80^

Since the reduction of silver and production of O_2_ gas necessitate the reformation of one BO from two NBO in the glass network,^11,17^ the Ag^0^ production and Ag nanoparticle (AgNP) yield are inherently correlated to the number of NBOs. Phosphates intrinsically start out with one more NBO than silicates, and most phosphates studied are at least metaphosphates (Q^2^ chains only), with additional *Q*^0^ and *Q*^1^ species, which have more NBOs than the disilicates and metasilicates reported to create AgNPs during the melt-quench and/or annealing process.^11,80^ Despite the larger number of NBOs in our glasses and annealing for 3 h at 1.07*T*_g_, without irradiation, we do not observe AgNPs, even in the presence of cerium, which should encourage Ag NP formation.^7,76^

Other authors have found a less-than-clear trend between AgNP yield and number of NBOs as well: fewer NBOs can still result in the same intensity from silver NP when the components of the glass matrix are varied, such as by the introduction of Al_2_O_3_.^80^ Although the number of NBOs is clearly a factor, we believe the type of NBO to be more important for the production of AgNPs.

The following structural assumptions are based on our previous NMR study of Ag-doped glasses,^17^ with the elemental analyses of these glasses (Table S1) matching those of our previously studied glasses. Furthermore, the Raman spectra in Figures S1–S5 agree with our previous NMR and Raman data.

In Figure S3a, the normalized Raman spectra of the PF20 sample have only slight differences between the four conditions: undoped, Ce-doped, Ag-doped, and Ag,Ce-codoped. The largest differences are in the high-frequency region. When Ag^+^, Ce^3+^ and Ag^+^, Ce^3+^ are added, the sharp *Q*^2^ peak, ∼1150 cm^–1^, decreases in intensity, while the intensity below 1125 cm^–1^ (shorter *Q^n^* units) increases for the two Ce-containing samples. This is in agreement with the addition of cations to the phosphate network reducing the total network connectivity and creating NBOs. The main difference between the dopants is the magnitude of intensity changes, where the presence of Ce^3+^ appears to encourage the formation of more *Q*_x_^1^ units, where x = Na^+^, Al^3+^, or Ce^3+^, as Ce^3+^ forms 3 times as many NBOs as Ag^+^. Finally, the presence of both Ag and Ce reduces the *Q*^2^ intensity more than cerium alone, again likely because of the higher combined cationic strength of Ag^+^ and Ce^3+^. The normalized Raman spectra for the PS10 set of samples shown in Figure S3b have larger differences than the PF20 spectra. This result is consistent with our conclusion in Section 3.2.2, where the optical properties of cerium were found to be more sensitive to Na_2_SO_4_ addition, indicating a stronger interaction between cerium and the PS matrix. The sulfate-related Raman peak intensity changes do not reflect the EDX results: Ag^+^-doped, Ce-doped, and Ag,Ce-codoped samples have 7.65, 6.04, and 5.21 mol % SO_3_, respectively, yet only the Ce-doped sample sulfate peak is sharply attenuated. There are two explanations for this discrepancy: first, Raman is only semiquantitative and the cross section of sulfate changes between dopants, and, second, the *Q*^1^ peak is hidden below the SO_4_^2–^ peak. When cerium is added, a unique phenomenon occurs: *Q*^1^ units decrease substantially, while the *Q*_Al_^1^ peak, ∼1065 cm^–1^, intensity increases. However, since no Al is being added or subtracted from the system and Ce^3+^ has a greater electronegativity than Na^+^, we assign the growth of the ∼1065 cm^–1^ shoulder to *Q*_Ce_^1^. Thus, we conclude that cerium strongly favors bonding with *Q*^1^ units in the PS glasses, in addition to converting *Q*^2^ units into *Q*_Ce_^1^ units—these two factors appear to lead to the noticeably larger peak at ∼1065 cm^–1^. Finally, silver addition appears to reset the *Q*^1^ units in the Ag,Ce glass while reducing the peak intensity assigned to *Q*_Al/Ce_^1^; we will discuss this more below. In Figures S4 and S5, the Ce- and Ag,Ce-doped glasses have few discernible differences as compared to the PF series, but more noticeable differences exist in the PS series. Only at high AlF_3_ addition, PF15 and PF20, does the introduction of silver result in lower intensity for the *Q*^2^ peak but no discernible changes elsewhere in the whole series. On the other hand, in the PS glasses, the same frequency region assigned to *Q*^2^, >1165 cm^–1^, increases in intensity for PS10–PS15, while the peaks assigned to *Q*^1^ and *Q*_Al/Ce_^1^ increase and decrease, respectively (the exception is PS15). Throughout our study, the optical properties exhibited opposite trends when silver was added: transmittance shifted to longer wavelength in PF, shorter in PS; emission intensity increased in PF, lowered in PS; lifetime increased for PF, decreased for PS; Ag clusters formed only in PS after irradiation but never formed in PF. It is likely that the opposite optical and structural trends are related to the fact that the intensity in the *Q*^1^ and *Q*^2^ regions has opposite trends in the two series as well.

**Figure 10:**
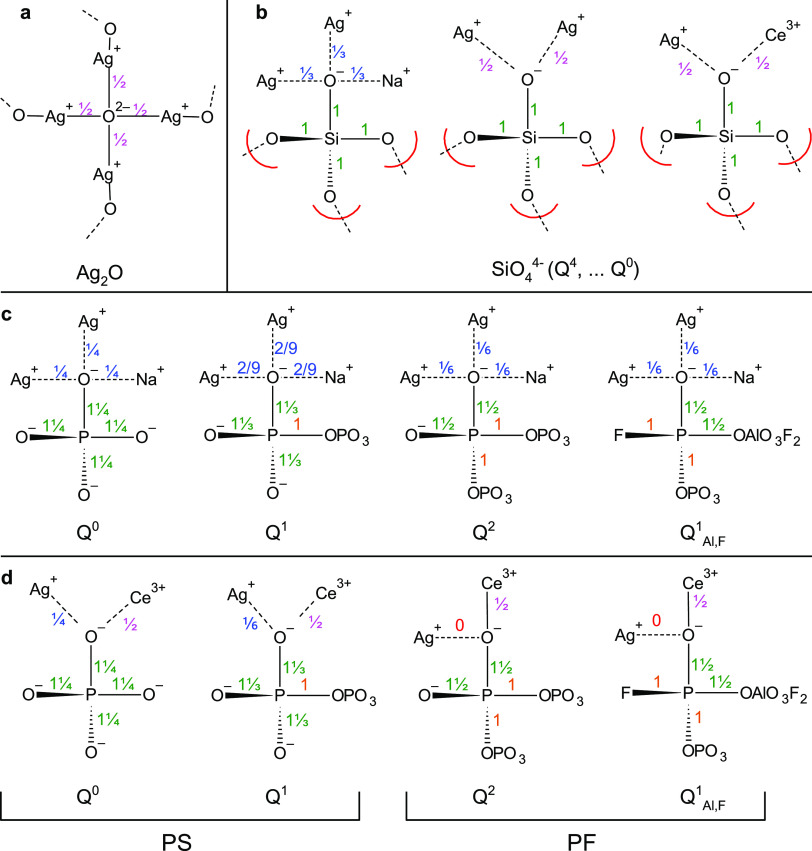
V.U. theory of various Ag-containing glass species: (a) crystalline Ag_2_O; (b) Ag–Ag and Ag–Ce interactions in all *Q^n^* units in silicates; and (c) Ag–Ag interactions and (d) Ag–Ce interactions in different *Q^n^* units in PF and PS glasses. Molecular structures are based on our previous structural studies of Ag-doped FP and SP glasses.^17^ See the text for more explanation.

We now turn to the valence unit (V.U.) theory in Figure 10 to explain the difference between silicate and phosphate NBOs, as well as the difference between phosphate groups found in the PF and PS glasses. V.U. theory, put forth from Bunker et al. and expanded upon by Brow et al., states that the valence of each high-field-strength cation be equally shared among each bond, the oxygen V.U. must add up to two, and the modifying cations are more flexible and have 0.1–0.4 V.U.^81,82^ Thus, we obtain one V.U. between each Si–O bond in Figure 10a, while in Figure 10b, we place one V.U. on either side of a bridging P–O–P bond, with the final fifth electron equally shared among the remaining NBOs in the phosphate tetrahedra. The Al^3+^ is expected to be mostly 6-fold-coordinated and fluorine can possess a maximum of one V.U.^17^ Finally, we assume that cerium and silver are normally in octahedral and tetrahedral coordinations; however, silver can also have only two bonds as in the case of Ag_2_O.^83^ We show possible Ag^+^ and Ce^3+^ interactions in Figure 10b–d, which are likely to lead to AgNPs, such as Ag^+^-clustering and Ag–Ce interactions.

In Figure 10a, we have drawn the crystal structure of Ag_2_O, where each Ag^+^ only has two oxide neighbors. Interestingly, the Ag–O bond has been shown to possess some covalency in Ag_2_O and Ag_2_SO_4_ due to the d-orbital contribution, unlike the Ag^+^···O^–^–P bond in AgPO_3_.^17,83,84^ We are assuming that Ag_2_O-like regions encourage Ag^+^ reduction since Ag_2_O is photosensitive and decomposes to Ag^0^ at 305–325 °C.^85^ Silver is a truly unique element in glass science: despite its relatively large ionic radius, 100 pm when tetrahedral, silver is more mobile than Li^+^ (ionic radius of 59 pm) and has a unique clustering behavior.^86,87^ Perhaps, this extra mobility is because of silver’s ability to create a structure-like crystalline Ag_2_O with only two oxide bonds, O–Ag–O, thereby statistically increasing the likelihood of the necessary complete bond-breaking required for ion mobility. Depending on its second-nearest neighbors, the Ag–O bond can shift its bonding character from more ionic to more covalent, similar to what has been reported for the Ce–O bond depending on Ce^3+^/Ce^4+^.^74^ This possible modifier–former duality may explain the reason that Ag^+^ can so easily cluster in the interstices of typical glass networks *before* reduction to Ag^0^.^11,88^ Since in oxide glasses former–former or modifier–former connectivity is favored over purely modifier–modifier association, cations that can act as both modifier and former, i.e., have both possible ionic- and covalent-bonding characters, might more easily cluster. This modifier–former duality is not unique to silver; ZnO, PbO, and MgO are other intermediates that can convert between being a network modifier or network former depending on the composition of the glass.^89−91^ This relatively common duality suggests that the bonding character is determined by second-nearest neighbors, and we should look at the character of the glass network in predicting the character of Ag–O bonding.

Let us examine the bonding in Ag^+^-doped and Ag^+^,Ce^3+^-codoped silicate glasses in Figure 10b. We observe that the Ag^+^–O^–^ bonding is more like that of Ag_2_O crystals, especially in the case of either Ag^+^–Ag^+^ and Ag^+^–Ce^3+^ interactions, where 1/2 V.U. is shared between the silver and oxygen. Another important distinction is that the *Q^n^* distribution in silicates does not affect the electron density on the oxygen of the NBO: the four electrons of Si^4+^ shared equally among the BOs and NBOs in *Q*^0^···*Q*^4^ in silicates (as shown by the red lines at the end of each oxide bond). Even when bonding in aluminosilicates is described, it is assumed that the AlO_4_^5–^ tetrahedra will be charge-compensated by a modifier, not that the SiO_4_^4–^ will charge-compensate AlO_4_^5–^ tetrahedra. This is unlike PO_4_^3–^ tetrahedra, which can possess the electron density per P–O bond to charge-compensate AlO_4_^5–^ tetrahedra in pure AlPO_4_.

Figure 10c demonstrates the difference in phosphates, where in a Ag^+^-doped glass, *Q*^0^ has Ag^+^–O^–^ bonding most similar to crystalline Ag_2_O in Figure 10a, while higher *Q^n^* groups result in progressively more ionic bonding between silver and oxygen. The addition of AlF_3_ has antagonistic effects, where Al–O–P bonding could lead to more covalent Ag^+^–O^–^ interactions, while P–F bonding has the inverse effect. This is exactly in line with our observations regarding Ag_m_^*x*+^ cluster formation. We believe the PS matrix to encourage more covalent Ag^+^–O^–^ bonding than the PF matrix, and this results in Ag_m_^*x*+^ cluster formation only in the PS matrix. In addition to the *Q*^0^ + *Q*^1^ units preferred for the formation of covalent Ag_2_O-like regions, the Ag^+^···O^–^–S bond was shown to be more covalent than the purely ionic Ag^+^···O^–^–P bond in Ag_2_SO_4_ and AgPO_3_, respectively.

The differences in Ag^+^–O^–^ bonding in the presence of cerium in Figure 10d demonstrate furthermore that *Q*^0^ and *Q*^1^ result in more covalent Ag–Ce interactions, while *Q*^2^ and *Q*_Al,F_^1^ can have Ce–O interactions with only minimal or purely ionic interaction with Ag^+^. Overall, the consistently different behavior of silver doping in these two glass series comes from the fact that silver acts as a glass modifier in PF glasses but can behave more covalently in PS glasses.

We conclude that when the glass matrix is composed of mostly *Q*^2^ and *Q*_Al_^1^ units as in the PF series, the addition of any cation behaves as expected—the creation of ionic NBOs, which results in more intensity in the *Q*_x_^1^ frequency region (x = Na^+^, Al^3+^ or Ce^3+^). However, when the structure has more *Q*^0^ and *Q*^1^ units, cerium bonds preferentially with *Q*^1^ units, resulting in a noticeable increase in *Q*_Ce_^1^ units. This agrees with the V.U. bond theory scheme, which shows more electron density being available for charge compensation of the Ce^3+^ in the shorter-chain phosphate groups.

In the Ag,Ce-doped samples, the Ag appears to convert *Q*_Al/Ce/Ag_^1^ back into *Q*^1^, perhaps because the covalent Ag–O interaction observed in Ag-doped PS glasses (as shown in our previous study, ref (17)) results in less electron density to be shared with the Ce^3+^. Overall, the interaction between Ag and Ce in the PF glasses appears to be limited, and any changes are due to there simply being more cations in the glass; this is also born out further by the lack of silver nanoparticles forming, despite the presence of Ce. On the other hand, due to the bonding preference of both Ag and Ce with *Q*^0^ and *Q*^1^, the Ag–Ce interactions affect the cerium bonding and optical properties in a very different way, along with encouraging nanoparticle formation. This matches well with the V.U. structures shown in Figure 10, where short-chain phosphates have sufficient electronegativity to form a stable Ag–O–Ce bond, while *Q*^2^ or *Q*_Al_^1^ can only form ionic bonds or an NBO with a single cation.

With that in mind, we now may explain the differences in cerium’s optical properties in the PF vs PS glasses upon Ag addition. In Figure 2, the Ag shifts the Ce^3+^ absorption cutoff to shorter wavelengths in the PF series, yet to higher λ in the PS series. As Duffy and Ingram and our previous paper^17^ discuss, when the orbital overlap between a color center and oxygen is more covalent, i.e., more former-like, a red shifting of the glass’ absorption edge occurs, while a more ionically bonded M^+^ results in a blue shift. This agrees with the trend we observe for PF (ionic Ag^+^) and PS (more covalent Ag^+^) glasses, but, surprisingly, the bonding environment of the silver in the PF and PS glasses also appears to influence the absorption cutoff of the Ce^3+^. Although a broad Ce^4+^ band should appear whose tail extends into the violet region, the shapes of our transmittance and absorption curves do not reflect the Ce^3+/4+^ ratio changing consistently when Ag is introduced. Ag^+^ absorption does not follow optical basicity trends^17^ and silver’s interaction with oxygen appears to change the degree of covalency of the Ce–O bond. We hypothesize that silver’s ionocovalent bonding character in the glasses dictates that of Ce–O, and therefore, the Ce^3+^ absorption cutoff wavelength is also influenced. Of course, this can also be considered in terms of the Ce^3+^/Ce^4+^ ratio, where a more covalent Ag–Ce interaction results in the stabilization of the more covalent Ce^4+^ and shifts the cutoff to shorter wavelengths.

**Figure 11:**
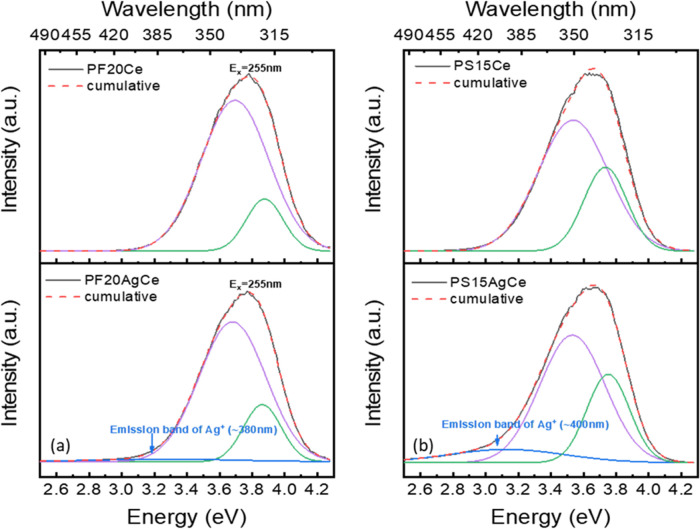
Gaussian deconvolution of the PL spectra of Ce-doped and Ag,Ce-codoped (a) PF20 glass and (b) PS15 glass.

If we now turn our attention to Figure 11 (deconvolution of Figure 5), we are surprised at how indistinguishable the Ag^+^ emission is from the main cerium peak in the codoped glasses, despite being excited at the expected excitation energy for Ag^+^ (255 nm). We know that silver remains in the form of Ag^+^ in these glasses (even in glasses with AgNPs, 50% of Ag is expected to remain as Ag^+^ ^11^) and the Ag^+^ fluorescence must be hidden in the tail of the Ce^3+^ peak between 400–450 and 450–500 nm in the PF and PS glasses, respectively. Indeed, other researchers have had similar challenges and also assigned the “shoulder” or “tail” to Ag^+^.^9^ In the codoped glasses, the Ag emission is less intense in the PF glasses compared to that of the PS glasses, while the main cerium emission peak has the opposite trend (PF > PS) compared to Ce-only compositions.

There are three factors that we might expect to dictate the Ce^3+^ emission intensity: the ionocovalent character of the Ce–O bond, cross-relaxation between Ag^+^ and Ce^3+^, and the ratio of Ce^3+^/Ce^4+^. Cross-relaxation between cerium and silver is the easiest factor to explore further. Our observation that the higher Ce^3+^ emission and relatively lower Ag^+^ emission following Ag doping of the PF glasses (Figure 5c) implies the energy transfer from Ag^+^ to Ce^3+^ in these glasses (which have more ionic Ag–Ce interactions). The Ce^3+^ emission from a 255 nm excitation in PS glasses (Figure 5d) remaining mostly unchanged following Ag doping suggests more covalent Ag–Ce interactions and cross-relaxation from Ce^3+^ to Ag^+^.

Differences in the fluorescence intensities of rare earth ions can be ascribed to local basicity and/or ion–ion interactions in addition to changes in oxidation state.^92^ In our present case, we do not think that the changes in the Ce^3+^ emission are driven only by a changing Ce^3+^/Ce^4+^ ratio, as when Ag^+^ is added to the PS glasses, the Ce^3+^ intensity from a 300 nm excitation decreases dramatically for all samples (Figure 4), yet there is no evidence of large Ag_m_^*x*+^ cluster formation for the nonirradiated samples (Figure 8b) nor is there clear Ce^4+^ formation after silver addition (Figure S6b). If the addition of Ag changes the Ce^3+^/Ce^4+^ ratio, then one could assume that the Ag^+^–O bonding pushes the Ce–O bond to be more covalent (Ce^4+^) in the PS glasses and more ionic (Ce^3+^) in the PF glasses.^74^

Although the concentration of the dopants is very similar in each glass composition, the silver equilibrium and resulting oxidation states are clearly different between the PF series and the PS series. We expect that the dopants behave like the ideal dilute limit where activity equals concentration; thus, the factor affecting the silver and cerium equilibrium constants is the oxygen activity of the glass. Unfortunately, we do not know the effective oxygen concentration, i.e., oxygen activity, but based on our calculations in Table S1, the [O] concentration is higher in the PS series than in the PF series and is highest for PS15. This is in line with there being more short-chain phosphate groups, and even *Q*^0^ units, in the PS series. It represents another reformulation of the concept of ionicity/covalency of the oxide bond, polarizability of the oxygen atoms, or *local* optical basicity of the glass matrix. Overall, the observation indicates a threshold oxygen concentration or activity, which promotes the formation of neutral silver.

During irradiation of the Ce-doped glasses, there is the expected trend in PL emission (Figure 6): the PL intensity decreases substantially, as it is related to the formation of Ce^4+^, due to the excitation of the 4f electron and stabilization of the newly generated (Ce^3+^)^+^ site.^9,72,93^ Similarly, the codoped glasses (Figure S9) also show decreasing PL intensity after irradiation. Only for the postirradiation data, PF and PS show the same trend in the presence of Ag^+^.

Finally, returning to Figure 9, we now understand the difference in emission lifetimes between PF and PS glasses upon silver introduction: silver increases the emission lifetime for Ce^3+^, especially in PF15 and PF20, while it decreases the lifetime in PS glasses. Based on our previous discussion, we posit that here too the way in which silver adds and interacts with the network and cerium affects the lifetimes. When the silver bonds more covalently, it pushes the Ce–O bond to be more covalent (Ce^4+^), decreases the Ce^3+^/Ce^4+^ ratio, and therefore decreases the Ce^3+^ lifetime. Conversely, the lifetime increases when silver acts as a modifier, where it encourages more ionic Ce–O bonding (Ce^3+^), increases the Ce^3+^/Ce^4+^ ratio, and thus increases the Ce^3+^ lifetime.

## 4 Conclusions

Cerium’s optical property trends can be adequately described using the optical basicity model. The cutoff wavelength and the emission band centers of Ce^3+^ shifted to longer wavelengths with decreasing fluoride and increasing sulfate content. Observed red shifts of absorption and emission peaks in cerium-doped glasses were found to be caused by increasing the optical basicity of the glass matrix. The shift for the cutoff wavelength in the Ce-doped PF series is larger than that in the PS series, which indicates that cerium does not prefer to associate with sulfate but is more strongly associated with *Q*^0^ and *Q*^1^ phosphate units.

After irradiation, the conversion of Ce^3+^ to Ce^4+^ results in a decrease of PL intensity and lifetime of Ce^3+^ in both PF and PS glasses. The addition of Ag increased the radiation resistance of the Ce-doped PF and PS glasses during irradiation. We only observed Ag_m_^*x*+^ clusters in PS glasses after irradiation, highlighting that in these PF and PS glasses, the highly ionic non-bridging oxygens promote the oxidized Ag^+^ species over the formation of Ag clusters or particles. This observation also shows that it is easier to generate Ag clusters in glass matrices with more covalent character, such as silicates.

The optical behavior of Ce^3+^ in PF and PS glasses was found to be influenced differently by the addition of Ag^+^ ions. The addition of Ag in Ce-doped PF glasses results in blue-shifting of the cutoff wavelength in transmittance spectra and enhanced PL intensity and lifetime of Ce^3+^ emission. The opposite was found in Ag,Ce-codoped PS glasses. We discussed three possible reasons for these variations: the ionocovalent character of the Ce–O bond, cross-relaxation between Ag^+^ and Ce^3+^, and the ratio of Ce^3+^/Ce^4+^. It is clear that all of these factors are interrelated, where the last two (cross-relaxation and valence) are all dependent on the ionocovalent bonding between cerium, silver, and the glass matrix. When the interactions of Ag^+^–O–Ce^3+^ are more covalent in PS glasses, the redox equilibrium shifts from Ce^3+^ to Ce^4+^; it promotes the formation of Ce^4+^ in PS glasses and thus weakens the PL intensity of Ce^3+^. On the other hand, the ionic Ag^+^–O–Ce^3+^ interactions in PF glasses encourage more Ce^3+^, enhance the PL intensity of Ce^3+^, and result in the opposite energy-transfer reaction.

Without irradiation or heat treatments above *T*_g_, these glasses show no propensity to form Ag–Ag pairs, and therefore, no Ag nanoparticles are present; yet, there are large variations in the intensity and cutoff of Ce^3+^ optical absorption. Our observations show that care must be taken when attributing some of RE luminescence enhancement and/or quenching to plasmon resonance of metallic silver; interaction of the RE species with Ag^+^ is another option. The type and magnitude of Ag^+^–RE interaction depend on the Ag^+^’s electronic environment. Overall, it is clear that there is a non-negligible influence of Ag^+^ on optically active ions: these effects must be taken into account and isolated when discussing the role of silver in the optical properties of such glasses.

## Supporting Information Available

Analyzed chemical compositions (Table S1), physical data (Table S2), supporting Raman scattering spectra (Figures S1–S5), supporting UV–vis transmission spectra (Figures S6–S8), and supporting PL data (Figures S9 and S10) (PDF)
