# Supplementary material for: Role of Ag+ Ions in Determining Ce3+ Optical Properties in Fluorophosphate and Sulfophosphate Glasses
Source: ACS Omega. 2021 Oct 26;6(44):30093–107. doi: 10.1021/acsomega.1c04933 (PMC8582266; doi:10.1021/acsomega.1c04933)
Supplement: Supplementary file 1 — ao1c04933_si_001.pdf [file ao1c04933_si_001.pdf]

Supporting Information for Publication

Role of Ag^+ ions in determining Ce^{3+} Optical Properties in Fluorophosphate and Sulfophosphate Glasses

Ru Zhou, Courtney Calahoo, Yicong Ding, and Lothar Wondraczek*

Otto Schott Institute of Materials Research, Friedrich Schiller University Jena, Fraunhoferstraße 6, 07743 Jena, Germany

*Email: lothar.wondraczek@uni-jena.de

This file contains supplementary Tables S1-S1 and Supplementary Figures S1-S10.

Table S1: Analyzed chemical compositions of the Ce-doped and Ag, Ce-codoped PF glass and PS glass series. The average error varies from 1–3 mol%.

PF Samples	[$NaO_{1/2}$] (mol%)	[$PO_{2/5}$] (mol%)	[AlF_3 + $AlO_{3/2}$] (mol%)	[F]/[Al]	[# of Na atoms]/[100 atoms]	[# of O atoms]/[100 atoms]
PF5-Ce	47.88	49.05	3.07	1.02	32.34	30.34
PF10-Ce	44.71	49.62	5.67	1.00	29.73	29.74
PF15-Ce	44.99	46.91	8.10	1.23	29.13	28.78
PF20-Ce	43.51	45.89	10.60	1.35	27.47	27.78
PF5-Ag,Ce	48.04	48.95	3.01	0.95	32.50	30.43
PF10-Ag,Ce	45.32	49.12	5.56	0.99	30.16	29.81
PF15-Ag,Ce	44.17	47.63	8.20	1.06	28.80	29.12
PF20-Ag,Ce	43.36	45.81	10.83	1.23	27.50	28.06
PS Samples	[$NaO_{1/2}$] (mol%)	[$PO_{2/5}$] (mol%)	[SO_3] (mol%)	[$AlO_{3/2}$] (mol%)	[# of Na atoms]/[100 atoms]	[# of O atoms]/[100 atoms]
PS5-Ce	44.66	43.23	1.24	10.87	29.65	33.60
PS7.5-Ce	45.68	41.40	1.98	10.94	29.93	34.48
PS10-Ce	46.33	39.78	3.11	10.78	29.77	35.73
PS12.5-Ce	47.05	38.52	3.81	10.62	29.88	36.49
PS15-Ce	47.61	37.45	4.19	10.75	30.03	36.91
PS5-Ag,Ce	44.65	43.24	1.32	10.79	29.61	33.68
PS7.5-Ag,Ce	45.73	41.12	2.24	10.91	29.83	34.77
PS10-Ag,Ce	46.52	40.15	2.67	10.66	30.12	35.25
PS12.5-Ag,Ce	46.81	38.07	4.54	10.58	29.38	37.17
PS15-Ag,Ce	47.89	37.02	4.43	10.66	30.09	37.22

Table S2: Density ρ, molar volume V_m, glass transition temperature T_g, Young's modulus E, Poisson ratio v and optical basicity $Λ_{th}$ of studied glasses.

Glass	Density (g/cm³) (±0.001)	V_m (cm³/mol)	Refractive index n_d (±0.0005)	T_g (°C) (±2)	E (GPa) (±0.7)	v (±0.003)	$Λ_{th}$	
							Nom.	Anal.
PF5-0.5Ce	2.550	39.854	1.516	293.0	39.9	0.291	0.455	0.454
PF5-0.5Ag-0.5Ce	2.558	39.861	1.517	287.4	39.6	0.283		
PF10-0.5Ce	2.583	38.997	1.509	314.2	42.3	0.287	0.440	0.449
PF10-0.5Ag-0.5Ce	2.587	39.070	1.511	312.3	42.7	0.283		
PF15-0.5Ce	2.614	38.192	1.508	333.5	45.3	0.282	0.425	0.447
PF15-0.5Ag-0.5Ce	2.617	38.281	1.510	337.0	46.2	0.283		
PF20-0.5Ce	2.629	37.633	1.507	369.9	52.7	0.275	0.410	0.439
PF20-0.5Ag-0.5Ce	2.639	37.781	1.510	370.7	52.7	0.275		
PS5-0.5Ce	2.645	39.502	1.520	413.6	52.1	0.282	0.4638	0.4525
PS5-0.5Ag-0.5Ce	2.661	39.476	1.521	418.1	52.9	0.280		
PS7.5-0.5Ce	2.646	39.880	1.521	414.6	51.4	0.286	0.4641	0.4579
PS7.5-0.5Ag-0.5Ce	2.652	39.910	1.523	416.9	51.4	0.280		
PS10-0.5Ce	2.643	40.304	1.524	414.8	50.6	0.283	0.4645	0.4583
PS10-0.5Ag-0.5Ce	2.652	40.285	1.525	411.6	50.1	0.287		
PS12.5-0.5Ce	2.637	40.774	1.526	412.6	49.6	0.292	0.4649	0.4585
PS12.5-0.5Ag-0.5Ce	2.650	40.691	1.526	414.4	49.9	0.290		
PS15-0.5Ce	2.635	41.185	1.527	410.6	48.2	0.293	0.4652	0.4589
PS15-0.5Ag-0.5Ce	2.642	41.191	1.528	417.9	48.1	0.291		

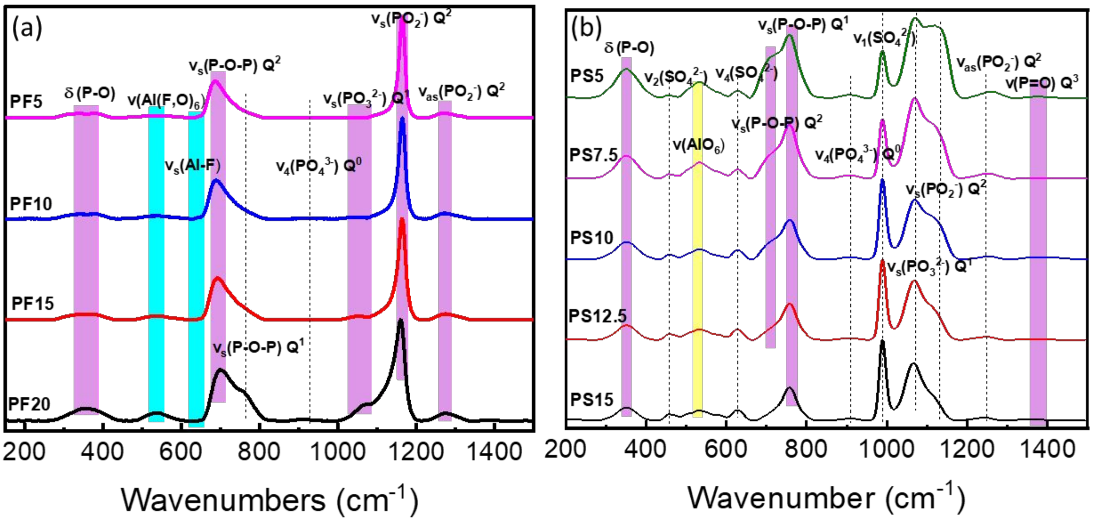

Figure S1:Raman spectra of Ce-doped (a) PF glasses and (b) PS glasses .

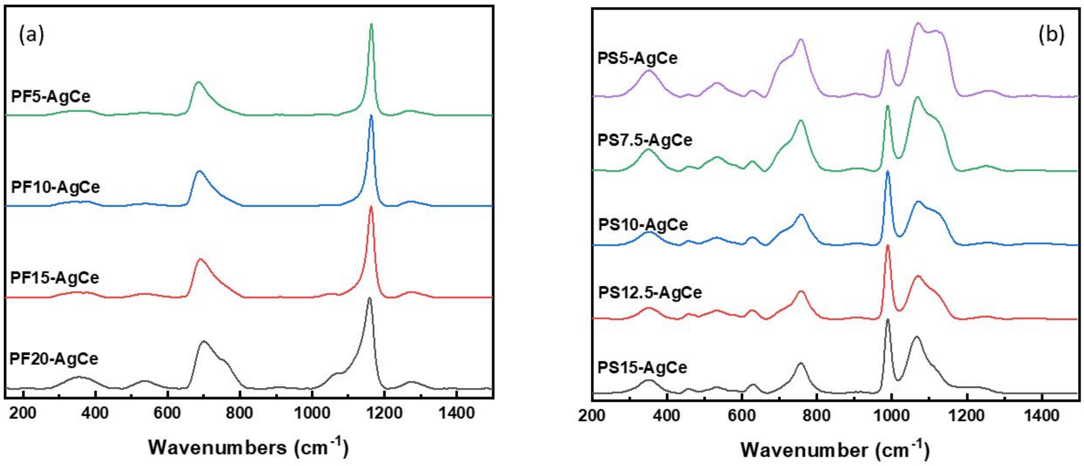

Figure S2:Raman spectra of AgCe-codoped (a) PF glasses and (b)PS glasses .

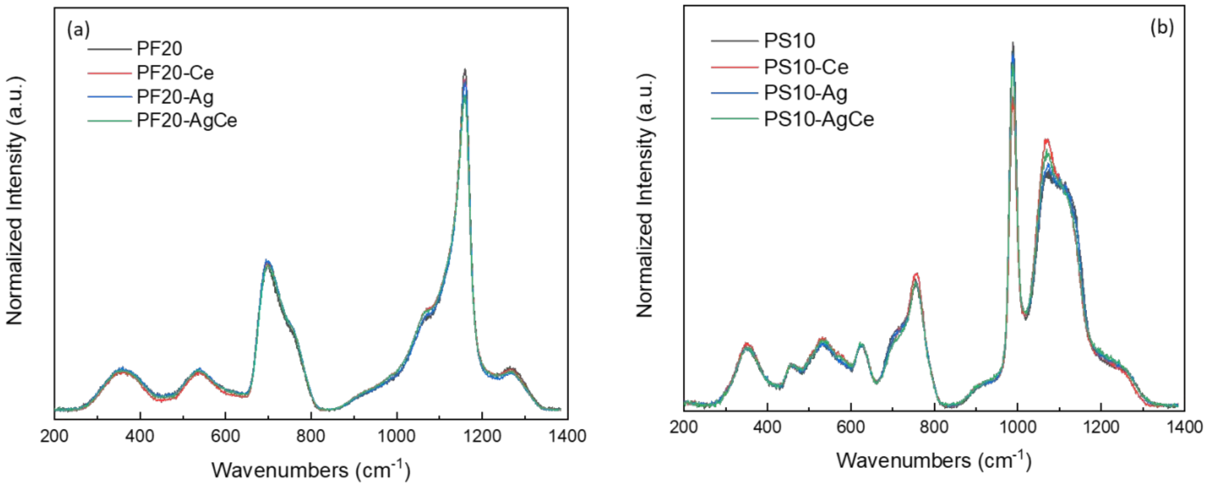

Figure S3:Normalized Raman spectra of (a) PF20 glasses and (b) PS10 glasses with different dopants.

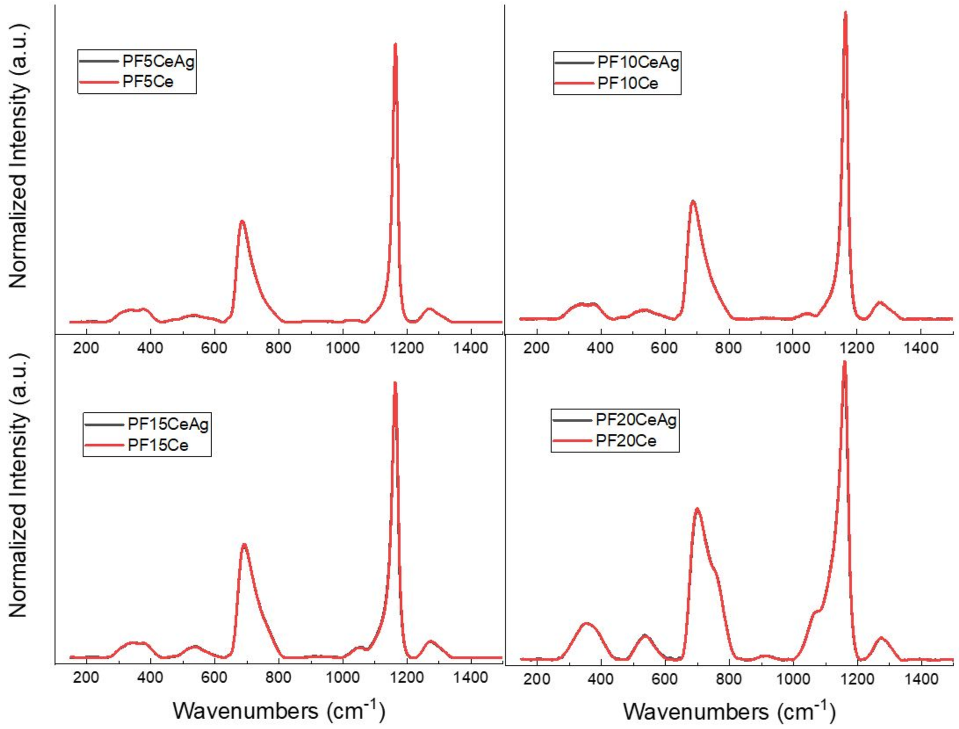

Figure S4:Normalized Raman spectra of PF glasses with single Ce and AgCe-codoping.

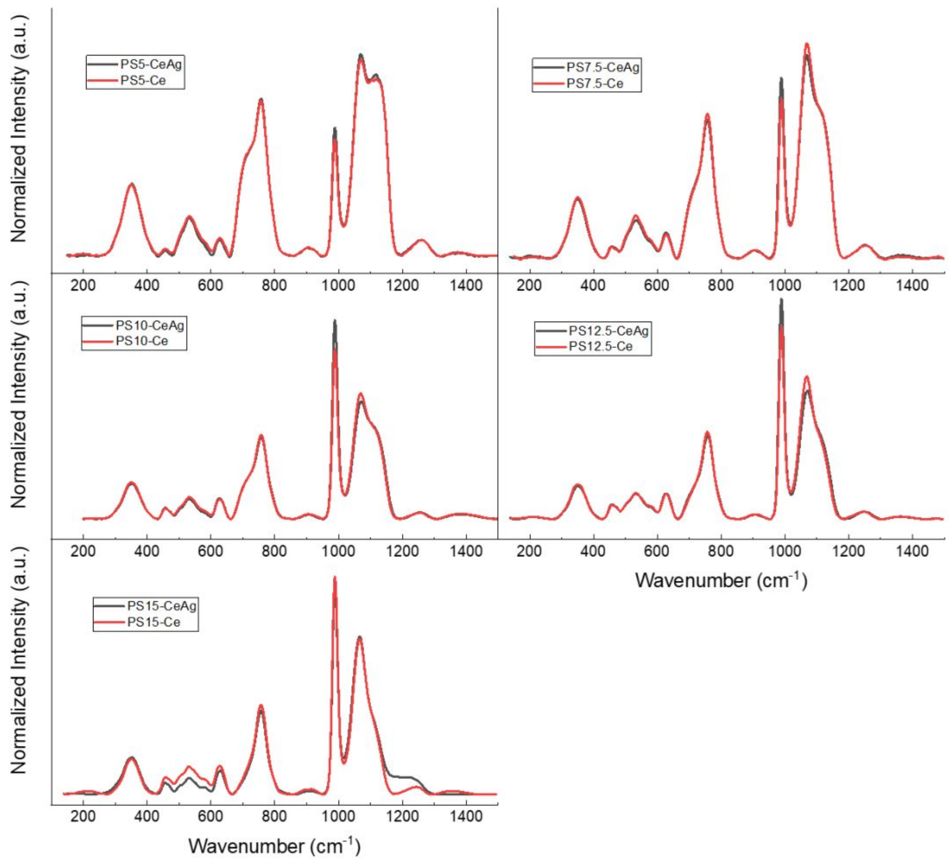

Figure S5: Normalized Raman spectra of PS glasses with single Ce and AgCe-codoping.

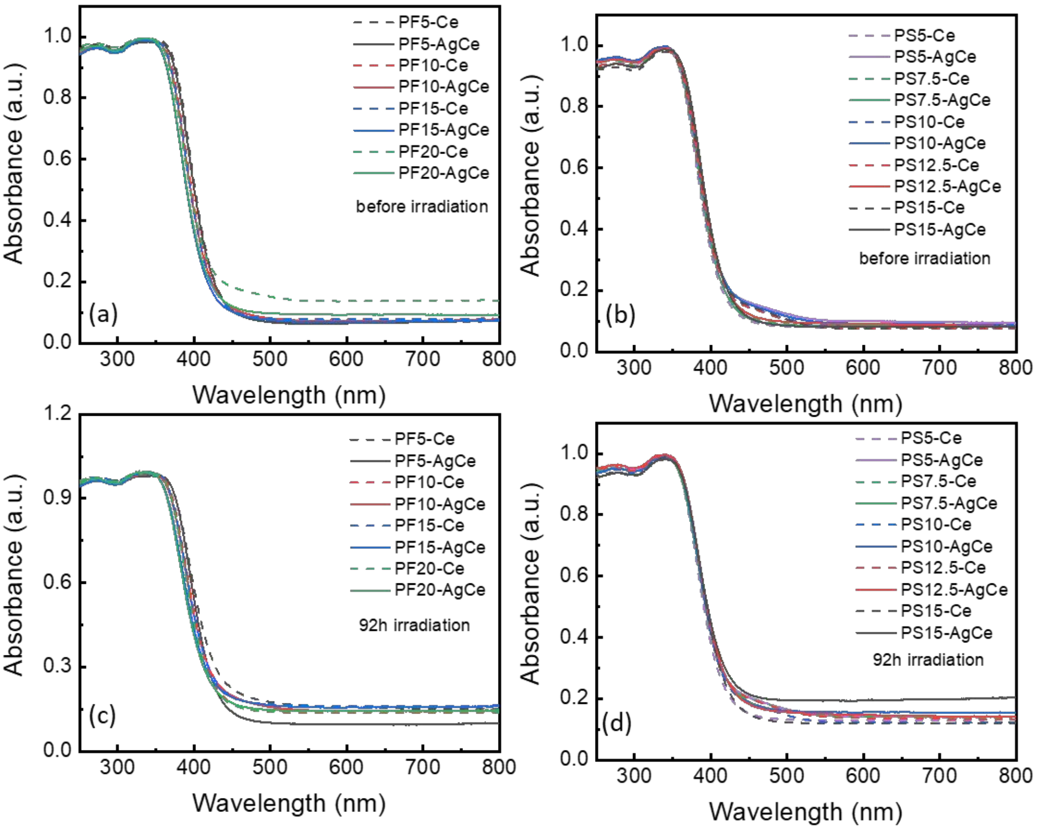

Figure S6:Absorption spectra of Ce-doped and Ag, Ce-codoped (a) PF glasses and (b)PS glasses before irradiation; the absorption spectra of Ce-doped and Ag, Ce-codoped (c) PF glasses and (d)PS glasses after irradiation.

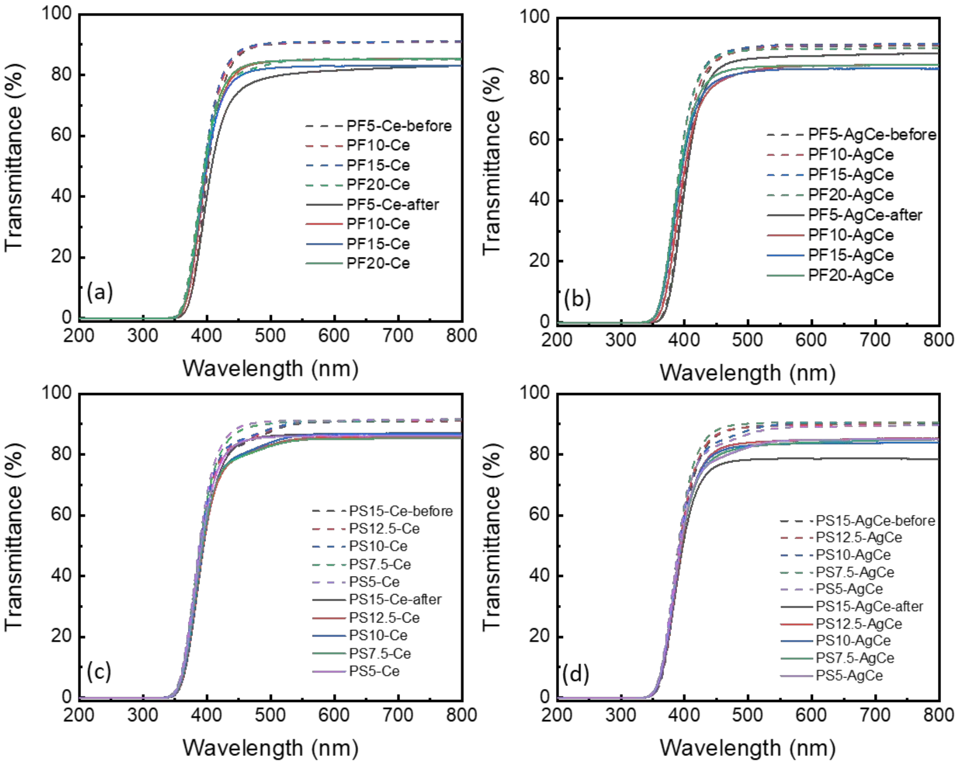

Figure S7:Transmittance spectra of (a) Ce-doped PF glasses, (b) Ag, Ce-codoped PF glasses, (c) Ce-doped PS glasses , and (d) Ag, Ce-codoped PS glasses before and after irradiation.

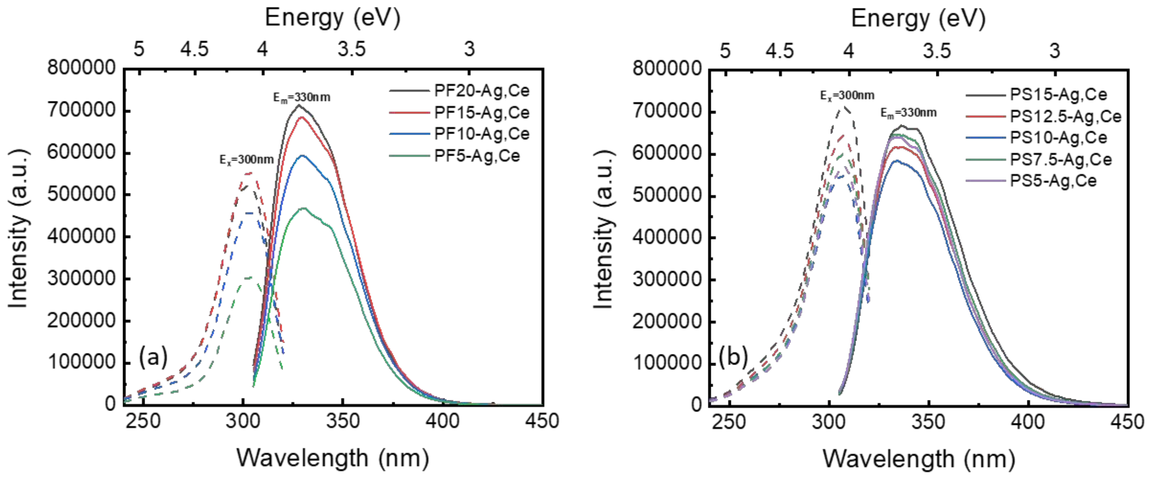

Figure S8: PLE and PL spectra of (a) Ag, Ce-doped PF glasses and (b) Ag, Ce-doped PS glasses under E_x=300 nm.

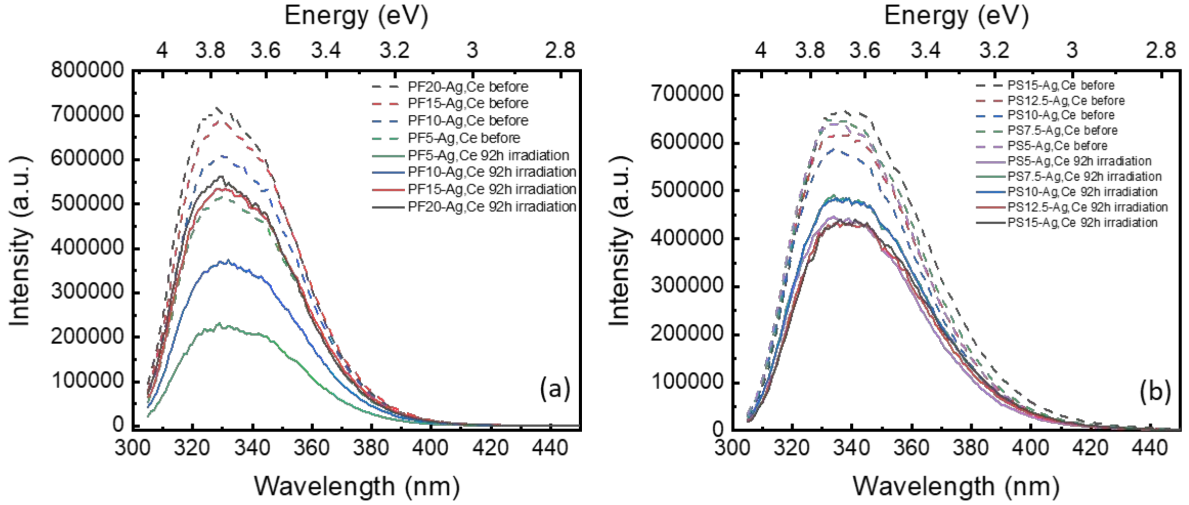

Figure S9: PL spectra of Ag, Ce-codoped (a) PF glasses and (b)PS glasses before and after irradiation under E_x=300nm.

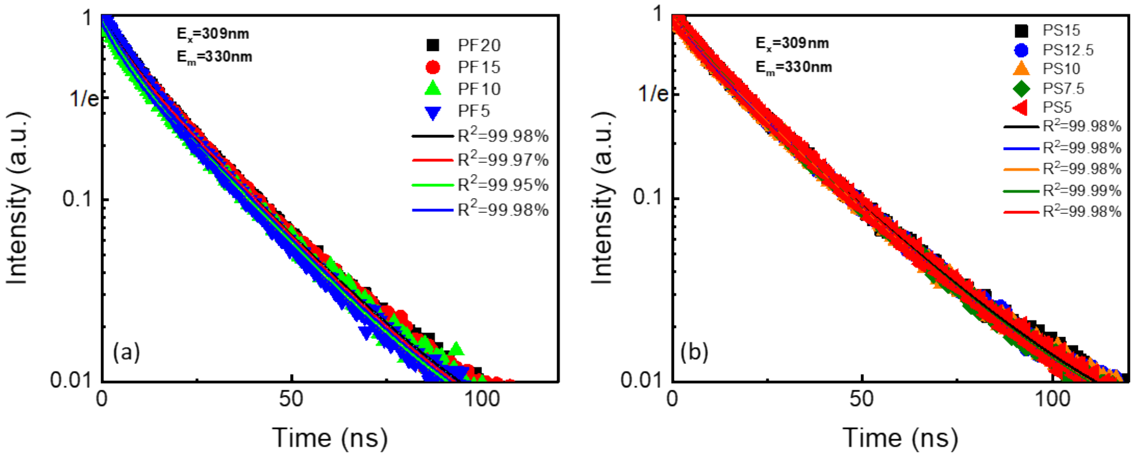

Figure S10: Luminescence decay curves of (a) Ce-doped PF glasses and (b) Ce-doped PS glasses (excitation and emission wavelengths as labelled).
